# Integrated bioinformatic analysis and machine learning developed a prognostic model based on mitochondrial function for acute myeloid leukemia

**DOI:** 10.3389/fimmu.2025.1597633

**Published:** 2025-10-23

**Authors:** Xingbiao Chen, Weijun Ling, Zhehan Yang, Xinyi Chen, Ziyuan Lu

**Affiliations:** ^1^ Department of Hematology, Guangdong Provincial Key Laboratory of Major Obstetric Diseases, Guangdong Provincial Clinical Research Center for Obstetrics and Gynecology, The Third Affiliated Hospital, Guangzhou Medical University, Guangzhou, China; ^2^ Department of Clinical Medicine, Guangzhou Medical University, Guangzhou, China; ^3^ Dongguan University of Technology - Conservatoire National des Arts et Métiers (DGUT-CNAM) Institute, Dongguan University of Technology, Dongguan, China; ^4^ Department of Biomedical Engineering, Guangzhou Medical University, Guangzhou, China

**Keywords:** mitochondrial-related molecular signature, acute myeloid leukemia, single-cell RNAsequencing, machine learning model, immune function

## Abstract

**Background:**

The disease burden of acute myeloid leukemia (AML) continues to pose a significant public health challenge globally. Mitochondria play a critical role in tumor development and progression by influencing bioenergetics, biosynthesis, and signaling pathways. However, the prognostic significance and therapeutic implications of mitochondrial function in AML warrant further investigation.

**Methods:**

We integrated mitochondrial gene expression data with bulk RNA sequencing to identify key mitochondrial genes associated with AML. A total of fourteen machine learning algorithms were employed, yielding 148 unique combinations. The best-performing model was utilized to develop a MitoScore, which was then combined with clinical variables to establish a MitoScore-based nomogram. Additionally, single-cell sequencing data were analyzed to assess the impact of key mitochondrial genes on immune cells. Samples were classified into low-risk and high-risk groups based on MitoScore, allowing for a comparative analysis of clinical features, biological mechanisms, copy number variations, tumor burden, immune infiltration, immune function, and drug sensitivity between the two groups.

**Results:**

Specific expression patterns of mitochondrial genes were observed in T cell subsets and at various developmental stages of AML. Samples were classified into low-risk and high-risk groups based on MitoScore. The high-risk MitoScore group exhibited a worse prognosis, with enriched biological processes and molecular pathways associated with immune response, a higher frequency of gene mutations linked to poor outcomes, increased immune cell infiltration, enhanced immune function, upregulated immune checkpoint gene expression, and greater sensitivity to cyclophosphamide and venetoclax.

**Conclusions:**

This robust machine learning framework underscores the potential of MitoScore as a tool for stratified prognostic assessment and personalized treatment planning in AML patients.

## 1 Introduction

Over the last three terms, the worldwide Burden of acute myeloid leukemia (AML)—the most substantial subtype of leukemia in adults—has escalated, impacting morbidity and mortality, remarkably, a few of the elderly and male populations, as mentioned through using the Global Burden of disease test (GBD) 2021 (1). This style highlights that the modern-day Burden of AML poses a significant public health challenge worldwide, necessitating collaboration among professionals, community physicians, and policymakers to develop customized healthcare approaches in the areas of diagnosis, treatment, and patient care. To optimize results for patients with AML, treatment regimens need to be meticulously tailored based on various factors, including age, genetic predisposition to leukemia, risk stratification, prior medical history, transplant eligibility, expected toxicity, and the patient’s medical profile and preferences (2). Similarly to age and comorbidities, the biology of the disease—in conjunction with cytogenetic and molecular changes—plays a crucial role in determining treatment regimens, responses, and survival prognoses (3). For example, the implementation of targeted chemotherapy has markedly improved survival outcomes for younger patients and those with adverse-hazard cytogenetics. Furthermore, allogeneic hematopoietic stem mobile transplantation remains a powerful restoration choice for patients with slight to horrible prognostic cytogenetics, but many old, frail, and significantly comorbid people are ineligible for transplantation (4). For this specific patient population, low-dose induction chemotherapy incorporating demethylating agents and venetoclax has been examined for efficacy in increasing the rate of complete response and extending median survival (5).

Additionally, several targeted therapies have recently gained acclaim for the treatment of AML, including ivosidenib and olutasidenib for IDH1 mutations (6, 7), enasidenib for IDH2 mutations (8), and gilteritinib and midostaurin for FLT3 mutations (9). Irrespective of these significant advances in drug development, some sufferers continue to experience refractory relapse, often due to the emergence of the latest clones or the activation of bypass signaling pathways following treatment, resulting in familiar healing outcomes that live suboptimal (10). Therefore, there may be a pressing need to understand potential healing dreams, optimize risk stratification structures, and refine custom-designed treatment techniques for AML.

A deeper understanding of the pathophysiological mechanisms underlying leukemia reveals that treatment failures can be attributed to the metabolic reprogramming and mitochondrial dysfunction of leukemia cells (11, 12). In 1924, Otto Warburg first defined that, in the presence of oxygen, tumor cells in fashionable depend on glycolysis for energy production, resulting in the accumulation of lactic acid. This metabolic method enables tumor cells to maintain their growth even as they acidify the surrounding microenvironment. Metabolic reprogramming serves as an adaptive mechanism for tumor cells inside the challenging conditions of hypoxia, nutrient deficiency, and immune surveillance, constituting the natural basis for tumor cell proliferation and the malignant phenotype (13). To satisfy their metabolic necessities, mitochondria perform several essential capabilities: 1) bioenergetics, which involves adjustments in ATP and NADPH production; 2) biosynthesis, encompassing the conversion of diverse vitamins from the microenvironment into vital additives for tumor cellular growth; and three) modulation of organic signaling pathways, thereby enhancing communication amongst tumor cells and other cells inside the microenvironment (14, 15).

Furthermore, extensive studies on the differentiation stages of leukemic cells have identified an unprecedented population of leukemic stem cells (LSCs) that act as the primary drivers of leukemia initiation and progression, contributing substantially to treatment resistance and relapse (16). LSCs exhibit remarkable mitochondrial morphology, energy metabolism, and regulatory mechanisms in contrast to hematopoietic stem cells and bulk cells. This particular mitochondrial profile underscores their reliance on mitochondrial oxidative phosphorylation (OXPHOS) (17). The mitochondrial dependence of LSCs additionally highlights their susceptibility to mitochondrial inhibitors, which can also serve as promising targets for novel drug development. Given the essential role of mitochondrial dependence in AML, the U.S. Food and Drug Administration (FDA) has approved several therapies for AML treatment, including the isocitrate dehydrogenase one inhibitor ivosidenib (6), the isocitrate dehydrogenase two inhibitor enasidenib (8), and the BH3 mimetic venetoclax (18). Furthermore, various mitochondrial inhibitors, including CPI-613, CB-839, IACS-010759, and ibrutinib, are currently under evaluation in clinical trials (19).

We examine the application of the Mitocarta 3.0 database, along with bulk RNA sequencing data, to identify key mitochondrial genes. We employed 14 tool-studying strategies across 148 combinations to select the simplest version, ultimately developing a Mitochondrial-related score (MitoScore). Moreover, we analyzed single-cell RNA sequencing (scRNA-seq) data to investigate the relationship between MitoScore and immune cellular populations, aiming to clarify the mitochondrial functional importance of immune cells within the leukemic microenvironment. Ultimately, we evaluated medical parameters, organic abilities, somatic variations, immune characteristics, and drug sensitivities across both high and low MitoScore groups. This comprehensive analysis aims to provide a theoretical resource for the prognostic rate of the MitoScore nomogram and to inform the development of custom-designed treatment strategies for AML.

## 2 Methods

### 2.1 Data collection

RNA sequencing data and clinical information for AML patients were obtained from The Cancer Genome Atlas (TCGA) database (https://portal.gdc.cancer.gov) and the Gene Expression Omnibus (GEO) datasets. The analysis included a total of 706 samples: 153 patient samples from TCGA-AML with associated survival data and 553 samples with survival data from the GEO dataset GSE37642. The GSE37642 dataset includes samples from both the GPL96 and GPL570 platforms. Additionally, we combined 116 AML patient samples and 69 standard bone marrow samples from the GSE30029 and GSE9476 datasets for differential expression gene (DEG) analysis using the limma package. Genes were considered significantly differentially expressed if the adjusted P value (adjP) was less than 0.05 and the fold change (FC) was greater than 1. In our analysis, we defined FC as the ratio of gene expression between two groups (e.g., disease vs. control) and logFC as the log-transformed value of FC, using a base two logarithm. Therefore, when FC > 1, it implies upregulation, and the corresponding log2FC is greater than 0. Conversely, when 0 < FC < 1, it represents downregulation, and log2FC is less than 0. This direct mathematical relationship is consistent with standard practices in gene expression analysis.

To focus on mitochondrial-related genes, we curated a list of 1,136 genes from MitoCarta 3.0, a comprehensive database of mitochondrial genes (https://www.broadinstitute.org/mitocarta). Genes not present in either the TCGA or GEO databases were excluded from our analysis. The final gene list is provided in **Supplementary Table S1**. We integrated the mRNA datasets from these sources and conducted a thorough screening process to identify differentially expressed genes (DEGs) associated with mitochondrial function. To visually represent the overlap between differentially expressed genes (DEGs) related to mitochondrial function and genes correlated with AML prognosis, we utilized the Venn diagram tool.

### 2.2 Construction of MitoScore signature

To construct the MitoScore signature, we employed Non-negative Matrix Factorization (NMF) using the NMF package to analyze the expression levels of 31 key mitochondrial genes across tumor samples. We integrated 14 distinct machine learning algorithms and evaluated 148 algorithm combinations (20, 21), including Random Survival Forests (RSF), Least Absolute Shrinkage and Selection Operator (LASSO), Elastic Net (Enet), Stepwise Cox Regression (StepCox), CoxBoost, Partial Least Squares Regression with Cox Proportional Hazards (PLSR-Cox), SuperPCA, Gradient Boosting Machine (GBM), Support Vector Machines for Survival Data (survivalism), Ridge Regression, Oblique Random Survival Forests (obliqueRSF), XGBoost, Conditional Inference Forests (CForest), and Conditional Inference Trees (CTree). A sequential approach was developed to identify the optimal prognostic variables through univariate Cox regression modeling.

The algorithms were applied to the overall AML cohort, which was divided into training and testing sub-cohorts in a 3:7 ratio. The best-performing model was constructed based on this split and further validated using both internal and external datasets. A MitoScore was calculated for each patient, and they were dichotomized into high- and low-score groups based on the median score. The prognostic significance of the MitoScore signature was assessed using Kaplan-Meier (KM) survival analysis. To confirm its predictive capability, we applied the MitoScore to the GSE37642 dataset, which contains survival information from an external AML cohort. Principal Component Analysis (PCA) and t-distributed Stochastic Neighbor Embedding (t-SNE) were employed to assess the predictive capability of the MitoScore. This methodological approach ensures a robust evaluation of the MitoScore as a prognostic tool for AML, providing a comprehensive assessment of its utility in predicting patient outcomes.

### 2.3 Construct a predictive nomogram

To enhance the predictive capability of the MitoScore signature, we developed a nomogram that integrates key clinical features with the MitoScore. These clinical features include age, sex, cytogenetic risk (cyto_risk; stratifying patients into favorable/intermediate/adverse groups based on ELN-defined chromosomal abnormalities), French-American-British classification (fab_code; classifying AML into M0-M7 subtypes by blast morphology and cytochemistry), and the MitoScore itself. The nomogram allows for the summation of scores from these variables for each patient, ultimately establishing a comprehensive survival prediction model. To validate the accuracy of the predicted survival rates at 1, 3, and 5 years, we generated calibration plots and time-dependent receiver operating characteristic (ROC) curves using the ggDCA and timeROC packages. Calibration plots were used to assess the agreement between observed and predicted survival probabilities, while time-dependent ROC analysis specifically evaluated the dynamic discrimination performance of the nomogram for 1-, 3-, and 5-year overall survival endpoints. The nomogram provides a robust tool for predicting patient outcomes by integrating both clinical and molecular characteristics, thereby improving prognostic precision. This approach ensures a thorough evaluation of the nomogram’s performance in predicting survival, facilitating more personalized and accurate prognosis for patients with AML.

### 2.4 ScRNA-seq data processing

We retrieved a scRNA-seq dataset (GSE235857) from the Gene Expression Omnibus (GEO) database, which includes samples from six patients with acute myeloid leukemia (AML) and six healthy controls. To integrate these samples, we employed the anchor-based integration approach provided by the Seurat R package (22). Following integration, we filtered the cells to retain only high-quality core cells for downstream analyses. Cells were excluded if they expressed genes detected in three or fewer cells or if fewer than 200 genes were detected per cell, as these criteria indicate low-quality data. For the retained core cells, gene expression levels were normalized using a linear regression model. We then identified the top 2,000 highly variable features through analysis of variance (ANOVA). PCA was performed on the single-cell samples, and the top 20 principal components (PCs) were selected for further study based on their contribution to the variance. To visualize and analyze the overall structure of the data, we applied the Uniform Manifold Approximation and Projection (UMAP) algorithm (23) for dimensionality reduction, using the top 20 principal components (PCs) as input. Cell type annotation was conducted using the SingleR R package (24), referencing the Human Primary Cell Atlas Data, Blueprint Encode Data, and Immune Cell Expression Data. Additionally, we utilized the CellMarker database (25) and previous studies to identify marker genes, thereby enabling manual annotation of distinct clusters. This comprehensive approach facilitated the accurate characterization of cell types within the scRNA-seq dataset, providing insights into the cellular heterogeneity associated with AML and its comparison to healthy states.

### 2.5 Identification of active subgroups

To evaluate which cellular subpopulations exhibit active mitochondrial function based on a gene set comprising 31 key mitochondrial genes, we utilized the R package “AUCell” to calculate an activity score for each cell. Specifically, we employed the AUCell_exploreThresholds function to determine the optimal threshold for identifying cells with significant mitochondrial activity. To visualize and identify subsets of cells specifically active in the context of these 31 mitochondrial genes, we colored UMAP embeddings of the cell clusters according to the Area Under the Curve (AUC) score obtained from each cell. This approach enabled us to pinpoint subpopulations with distinct mitochondrial activity patterns, thereby providing insights into the functional heterogeneity of mitochondria across different cell types within the scRNA-seq dataset.

### 2.6 Further analysis of T cell subgroups

Based on the active subpopulations identified through the AUCell analysis, we extracted the T cell subpopulations and performed re-dimensionality reduction and reclustering. This process resulted in the annotation of five distinct T cell subpopulations. To elucidate the molecular mechanisms underlying the progression of AML, we applied the Monocle 2 algorithm to conduct pseudotime trajectory analysis on these five T cell subpopulations. This analysis allowed us to infer the temporal ordering of cellular states and identify key transcriptional changes associated with AML progression. Additionally, we utilized CellPhoneDB v2.0 to investigate potential intercellular interactions among the five T cell subpopulations. By analyzing ligand-receptor pairs, we identified putative communication networks that may play a role in the dynamic changes observed within the T cell landscape during AML progression. This comprehensive approach provides insights into the dynamic changes and intercellular communication within the T cell environment, offering a deeper understanding of the immunological processes involved in AML progression.

### 2.7 Biological function and pathway enrichment analysis

To investigate the biological functions and pathway processes associated with the MitoScore, we conducted a series of enrichment analyses using the Kyoto Encyclopedia of Genes and Genomes (KEGG), Gene Set Variation Analysis (GSVA), and Gene Set Enrichment Analysis (GSEA). These analyses were performed using the R packages clusterProfiler, GSVA, and GSEABase. GSVA was employed to transform gene expression data from single-gene measurements into gene set enrichment scores, thereby assessing the extent of enrichment for each gene set within individual samples. This approach allows us to evaluate how pathways related to the MitoScore are represented across the dataset, providing insights into the functional implications of mitochondrial gene expression patterns. KEGG pathway analysis was used to identify significantly enriched pathways, while GSEA was applied to further explore the overrepresentation of specific gene sets in high- versus low-MitoScore groups. Together, these analyses provide a comprehensive understanding of the biological processes and pathways that are differentially regulated in association with the MitoScore, thereby elucidating the molecular mechanisms underlying its prognostic significance.

### 2.8 Mutation landscape analysis

To investigate the genomic landscape of AML patients, we obtained copy number variation (CNV) profiles using the TCGA Bioconductor package. Patients were categorized into subgroups based on the MitoScore model’s threshold, allowing for a stratified analysis of CNVs. We employed GISTIC2.0 to identify genomic regions exhibiting significant amplifications or deletions across the samples, thereby pinpointing specific somatic copy-number alterations associated with the MitoScore (26). Furthermore, we utilized the dplyr package to compare the frequency of common somatic mutations between patients with high and low MitoScores. To assess the tumor mutation burden (TMB), we calculated the total count of non-synonymous somatic mutations per megabase across the entire genome.

### 2.9 Assessment of immune microenvironment

To comprehensively evaluate the levels of immune infiltration and molecular characteristics, we employed a suite of bioinformatics algorithms, including ssGSEA (27), CIBERSORT (28), CIBERSORT-ABS (29), QUANTISEQ (30), MCPcounter (31), Xcell (32), and EPIC (33). These algorithms utilize distinct strategies to estimate the abundance of various immune cell subpopulations, providing a multi-faceted view of the tumor microenvironment. We further analyzed immune activity across six immune subtypes: the wound healing subtype (C1), IFN-gamma dominant subtype (C2), inflammatory subtype (C3), lymphocyte-depleted subtype (C4), immunologically quiet subtype (C5), and TGF-beta dominant subtype (C6) (34). The ssGSEA R package was used to calculate enrichment scores or relative abundances for various immune features by evaluating signature genes. This approach allowed us to quantify the presence and activity of different immune cell types within each sample. Additionally, we examined the expression patterns of 60 immune-related factors, including genes involved in antigen presentation, cell adhesion, co-inhibitory molecules, co-stimulatory molecules, ligands, and receptors. This analysis provided insights into the functional states of immune cells and their interactions within the tumor microenvironment. Moreover, we conducted an in-depth analysis of the relationship between immune checkpoint genes and the MitoScore. This investigation aimed to elucidate how mitochondrial activity correlates with immune checkpoint expression, potentially uncovering novel therapeutic targets for modulating the immune response in AML.

### 2.10 Investigating the importance of the MitoScore in the drug sensitivity analysis

To investigate the impact of MitoScore on treatment outcomes in AML, we calculated the IC50 values for commonly used chemotherapeutic agents using a custom algorithm and the pRRophetic R package. The pRRophetic package leverages gene expression and drug sensitivity data from cancer cell lines obtained through the Cancer Genome Project to develop statistical models. These models predict chemotherapeutic responses based on baseline tumor gene expression profiles, enabling the estimation of drug sensitivity in patient samples. We applied this approach to a melanoma dataset to calculate IC50 values for a panel of anti-tumor drugs. To evaluate differences in chemotherapeutic outcomes between patient groups, we compared the IC50 values between high-risk and low-risk groups using the Wilcoxon signed-rank test. The results were visualized using box plots, providing a clear comparison of drug sensitivities across the two groups.

### 2.11 Statistical analyses

For bulk RNA-seq data from the TCGA and GEO databases, we downloaded raw expression matrices in FPKM or count format where applicable. GEO datasets with Affymetrix platforms (e.g., GPL96, GPL570) were processed from CEL files using the RMA algorithm via the Affy package. Gene expression data were log2-transformed using log2(x + 1) to reduce skewness and stabilize variance. To enable cross-dataset comparisons and integration, we employed quantile normalization across all samples and utilized the ComBat algorithm from the Sva package to mitigate batch effects between different platforms. Genes with low expression (average counts <1 in over 50% of samples) were excluded before differential expression analysis. For single-cell RNA-seq data, we applied standard Seurat workflows, including log-normalization, feature selection, and scaling, before proceeding with downstream dimensionality reduction and clustering.

Prognostic modeling integrated multiple machine learning algorithms selected for their complementary capabilities: LASSO-Cox regression implemented feature selection through L1-penalized dimensionality reduction; Random Survival Forests captured non-linear interactions and complex dependencies; while XGBoost enhanced predictive accuracy in structured clinical data [CitationX]. Hyperparameter optimization employed systematic grid search within stratified 5-fold cross-validation (70% training subsample), evaluated by Harrell’s C-index. Overfitting mitigation was incorporated through survival-stratified cross-validation, temporal test set evaluation (30% cohort), and regularization, including L1-penalization (λ = 0.02) with early stopping (50-iteration patience).

All statistical analyses were conducted using R (version 4.3.3; https://www.r-project.org/). For comparisons between two groups, we employed the Wilcoxon rank-sum test, whereas for multiple-group comparisons, the Kruskal-Wallis test was used. Spearman’s rank correlation analysis was applied to calculate correlation coefficients. Fisher’s exact test or chi-squared test was utilized for comparing contingency tables and categorical variables. Kaplan-Meier survival analysis, accompanied by the log-rank test, was performed to compare prognostic outcomes between subgroups. Univariate and multivariate Cox proportional hazards regression analyses were used to estimate hazard ratios (HR) for various factors. Statistical significance was denoted as follows: *p < 0.05, **p < 0.01, ***p < 0.001, and ns for not significant.

## 3 Results

### 3.1 Preliminary screening of key mitochondrial genes

**Figure 1:**
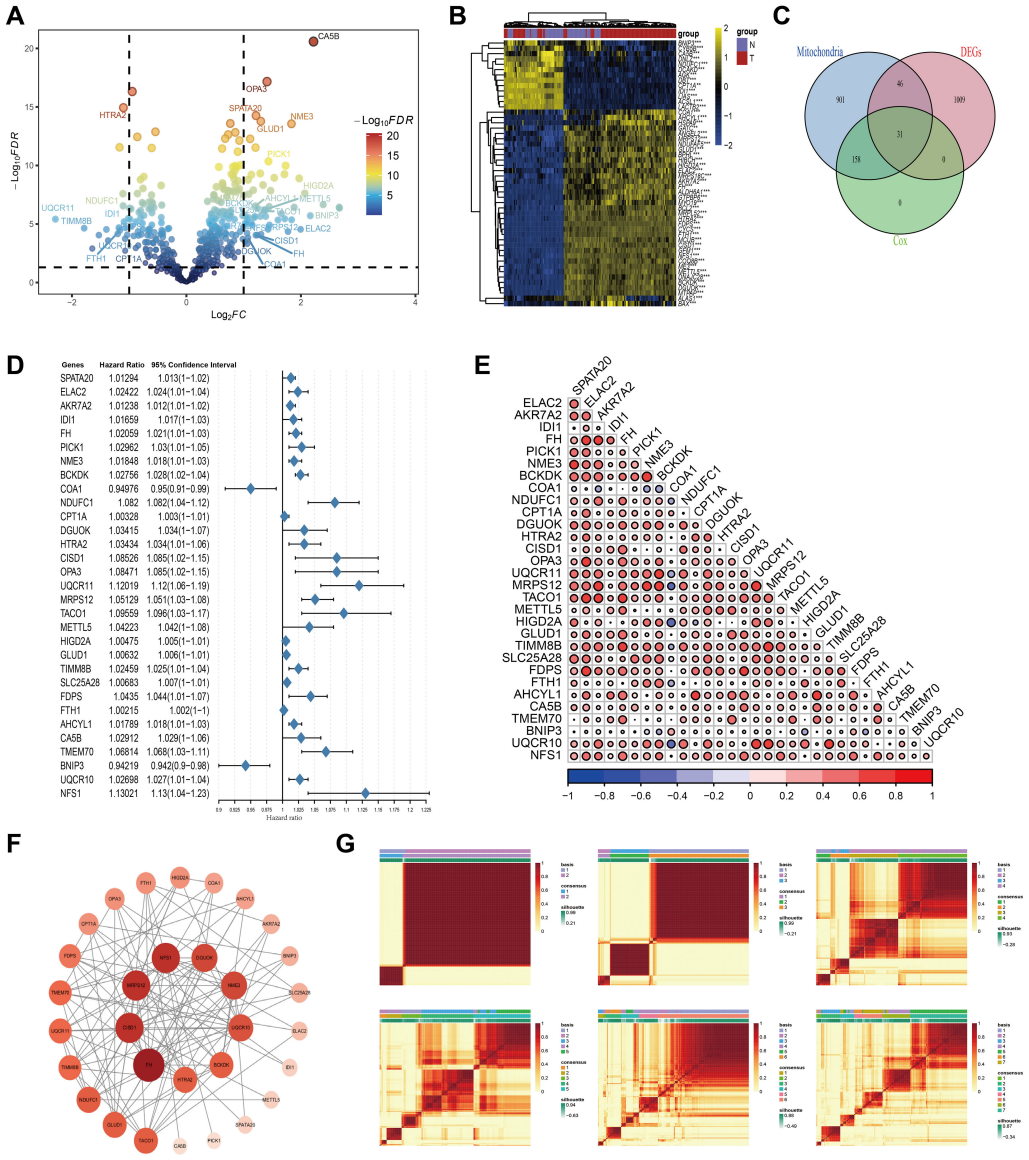
Preliminary screening of key mitochondrial genes. **(A)** Volcano plot of the differential expressed genes analysis in the AML and adjacent normal tissues. Mitochondria-related genes with prognostic value marked out. **(B)** Heatmap displaying the top fifty DEGs. **(C)** Venn plot of DEGs, prognostic genes, and mitochondria-associated genes. **(D)** Univariate Cox regression analysis of OS based on 31 key mitochondrial DEGs. **(E)** Correlation analysis results among 31 key Mitochondrial DEGs. **(F)** PPI Network Analysis Results of 31 key Mitochondrial DEGs. **(G)** Distinguishing two different subtypes using the NMF algorithm.

In this study, we acquired gene expression profiles and corresponding clinical data from TCGA and GEO databases. From the MitoCarta3.0 database, we compiled a comprehensive list of 1,136 genes known to be associated with mitochondrial function. We performed differential expression analysis to identify differentially expressed genes (DEGs) between normal tissue and AML samples (**Figures 1A, B**). Following this, we performed univariate Cox regression analyses on AML prognosis for genes associated with mitochondrial function. Using a Venn diagram approach, we identified a subset of differentially expressed genes (DEGs) that concurrently influence both mitochondrial processes and AML prognosis (**Figure 1C**). The first gene set represents differentially expressed genes (DEGs) identified by comparing AML samples to standard bone marrow controls using the limma package, with thresholds of an adjusted P-value < 0.05 and a fold change > 1. The second gene set comprises prognosis-related genes, which were screened using univariate Cox regression analysis in the TCGA-AML cohort (P < 0.05). The third gene set comprises mitochondrial-related genes curated from the MitoCarta 3.0 database, a comprehensive resource of genes with strong evidence for mitochondrial localization and function. The intersection of these three sets yielded 31 key genes that are (1) mitochondria-associated, (2) differentially expressed between AML and normal samples, and (3) significantly associated with patient survival. These genes formed the basis for constructing the downstream model. This rigorous selection process culminated in the identification of 31 key mitochondrial genes: SPATA20, ELAC2, AKR7A2, IDI1, FH, PICK1, NME3, BCKDK, COA1, NDUFC1, CPT1A, DGUOK, HTRA2, CISD1, OPA3, UQCR11, MRPS12, TACO1, METTL5, HIGD2A, GLUD1, TIMM8B, SLC25A28, FDPS, FTH1, AHCYL1, CA5B, TMEM70, BNIP3, UQCR10, and NFS1. A forest plot was constructed to illustrate the results of the univariate Cox regression analysis for these 31 genes within the TCGA_AML cohort (**Figure 1D**).

Additionally, we explored the interrelationships among these genes using a Pearson correlation heatmap (**Figure 1E**), where red and blue circles denote significant positive and negative correlations, respectively. The size and depth of color reflect the strength of the correlation. This analysis revealed strong co-expression patterns among multiple genes, suggesting coordinated regulatory mechanisms. To provide a comprehensive visualization, we employed the STRING database to generate a gene co-expression network, which was enhanced in Cytoscape (**Figure 1F**). In this network, node size and color intensity scale with connectivity degree (number of edges), identifying FH, MRPS12, CISD1, and NFS1 as core genes (highest connectivity) potentially central to mitochondrial regulation in AML. To refine our prognostic model for AML, we applied clustering analysis to the activity levels of the 31 characteristic genes, which revealed K = 2 as the most robust cluster (**Figure 1G**). The expression patterns of these genes were depicted through box plots and ridge plots (**Supplementary Figures S1A, B**). Moreover, we conducted a pan-cancer analysis to examine the expression levels and prognostic significance of these genes across various malignancies. This analysis offered insights into the broader expression landscape (**Supplementary Figure S2A**) and potential implications for patient outcomes across different cancer types (**Supplementary Figure S2B**).

### 3.2 Establishment of the MitoScore signature

**Figure 2:**
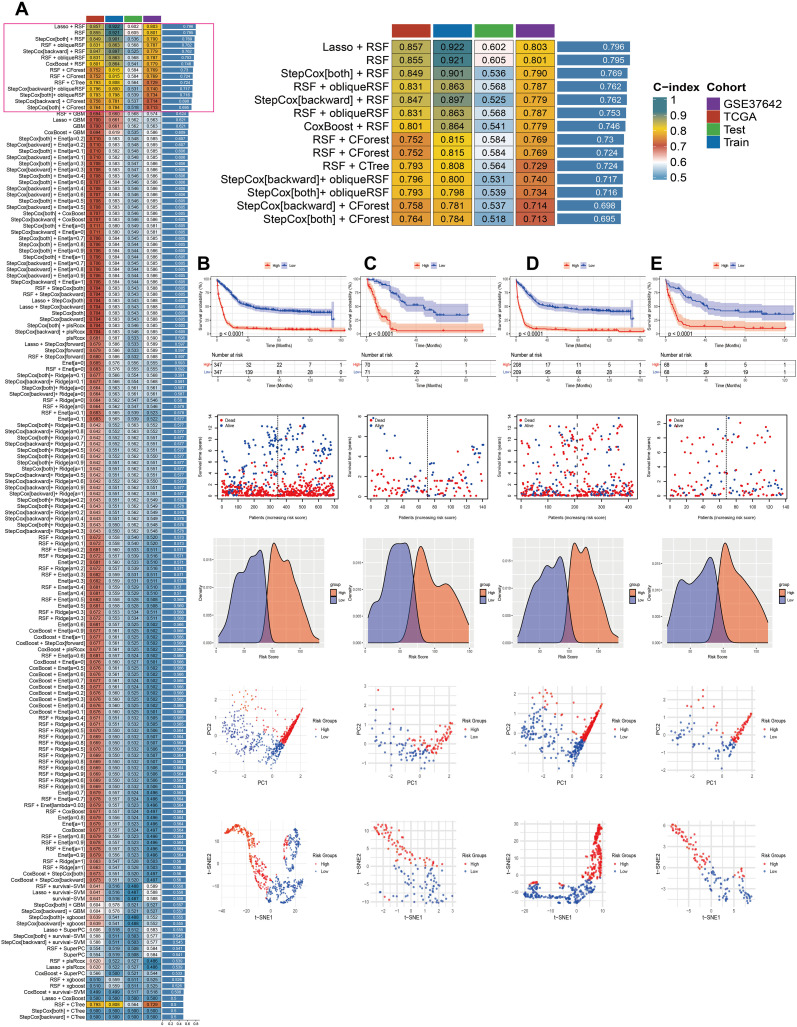
Establishment of the MitoScore signature. **(A)** Using 148 different combinations of 14 machine learning algorithms for MitoScore research, with C-index calculated for each model. **(B)** Survival curve of the total cohort. **(C)** Survival curve of the TCGA cohort. **(D)** Survival curve of the GSE37642_GPL96 cohort. **(E)** Survival curve of the GSE37642_GPL570 cohort. Survival status, density distribution, PCA analysis, and tSNE analysis between two different subgroups.

To construct and evaluate a robust prognostic model, we employed a suite of 14 machine learning algorithms, including RSF, Lasso, Enet, stepcox, CoxBoost, plsRcox, SuperPCA, GBM, survivalism, Ridge Regression, obliqueRSF, XGBoost, CForest, and CTree. We also explored combinations of these methods to enhance predictive performance. The average C-index was calculated for 148 algorithmic configurations across the entire dataset, as well as separate training and validation sets, to determine the optimal model configuration (**Figure 2A**). The MitoScore model, which emerged as the most effective, integrates the Lasso and RSF algorithms (**Figure 2B**). To validate the model’s performance, we applied it to external datasets, including TCGA_AML, GSE37642_GPL96, and GSE37642_GPL570. Patients were stratified into high-risk and low-risk groups based on the median MitoScore within each cohort. K-M survival analysis revealed that patients in the high-risk group exhibited significantly worse prognoses compared to those in the low-risk group (**Figures 2C–E**). A higher MitoScore was consistently associated with a poorer prognosis, demonstrating the model’s excellent predictive accuracy. Dimensionality reduction techniques, such as PCA and t-SNE, highlighted distinct clusters corresponding to the high-risk and low-risk patient groups, further supporting the model’s ability to distinguish between different risk profiles (**Figures 2C–E**). These findings underscore the clinical utility and prognostic significance of the MitoScore, positioning it as a valuable tool for personalized medicine in AML.

### 3.3 Construction of a prognostic nomogram based on MitoScore

A prognostic nomogram was developed based on the MitoScore to evaluate its predictive utility in patients with AML. Univariate and multivariate Cox regression analyses were performed to assess the independent and combined predictive power of the MitoScore. The nomogram integrates the MitoScore with clinically relevant features, including age, sex, cyto_risk, and fab_code, thereby providing a quantitative tool for predicting AML patient outcomes and supporting clinical decision-making (**Supplementary Figure S3A**). To estimate overall survival probabilities, the nomogram for TCGA AML patients incorporates these key variables. Calibration curves were used to validate the accuracy of the prediction model, demonstrating a strong concordance between predicted and observed survival probabilities at 1-year, 3-year, and 5-year intervals (**Supplementary Figure S3B**). The AUC values for both the MitoScore alone and the integrated nomogram at these time points exceeded 0.75, as determined by time-dependent ROC analysis, indicating high diagnostic performance (**Supplementary Figures S3C–G**). DCA further revealed that the nomogram model provides substantial net benefit across a wide range of threshold probabilities, enhancing its clinical applicability (**Supplementary Figures S3H, I**). These findings collectively demonstrate that the nomogram, which leverages MitoScore characteristics, exhibits excellent performance in predicting the prognosis of AML patients, thereby offering a valuable resource for personalized medicine.

### 3.4 Single-cell sequencing of the MitoScore model

**Figure 3:**
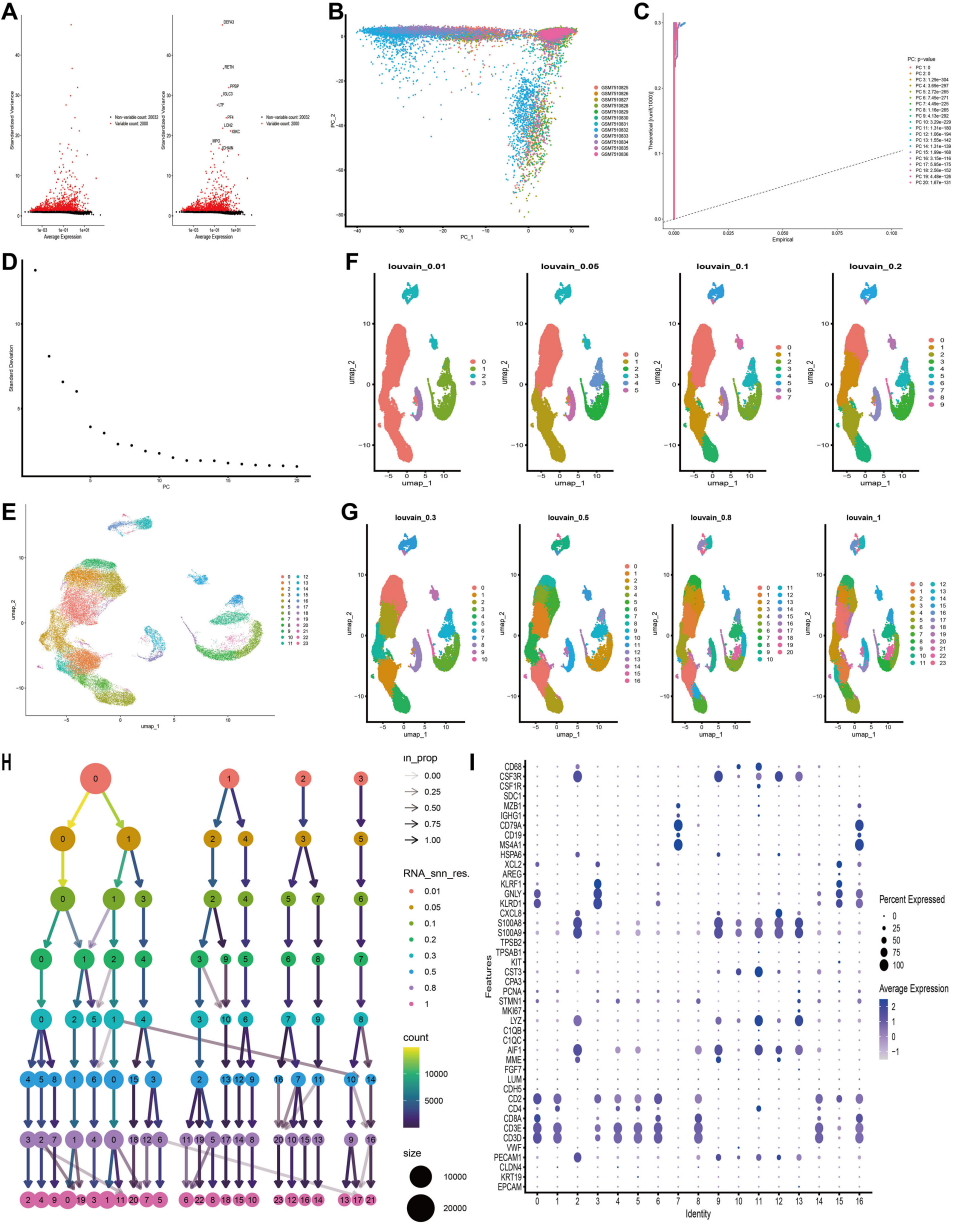
Based on single-cell RNA sequencing data, cell clusters with different annotations were identified, revealing high cellular heterogeneity in AML. **(A)** The variance diagram shows the variation of gene expression in all cells of AML. The red dots represent highly variable genes and the black dots represent non-variable genes. **(B)** PCA showed a clear separation of cells in AML. **(C, D)** PCA identified the top 20 PCs at P < 0.05. **(E)** The UMAP algorithm was applied to the top 20 PCs for dimensionality reduction, and 24 cell clusters were successfully classified. **(F–H)** Present the clustering outcomes of cell clusters at varying resolution levels. **(I)** Expression levels of marker genes for each cell cluster.

Initially, we filtered out unqualified cells, obtaining 39,783 core cells for subsequent analysis. We performed ANOVA on the genes within these core cells and identified 2,000 highly variable genes (**Figure 3A**). PCA was conducted on 12 single-cell samples, revealing a reasonable distribution of the samples (**Figure 3B**). In the PCA, we selected 20 principal components for further analysis, all of which had p-values < 0.05 (**Figures 3C, D**). Subsequently, we used the UMAP algorithm to classify the core cells into 24 cell clusters (**Figure 3E**). We further presented the results of cell clustering at different resolution levels using UMAP plots and dendrograms (**Figures 3F–H**). The signature genes for each cell cluster were displayed using bubble plots (**Figure 3I**).

**Figure 4:**
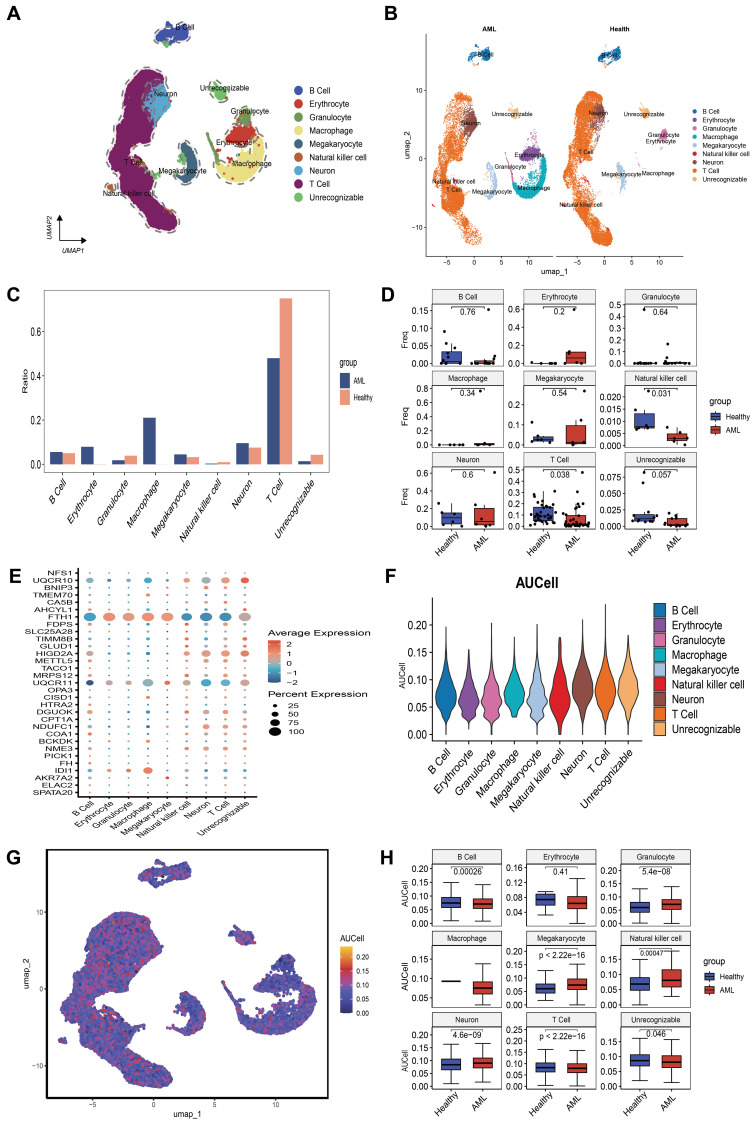
An overview of the single-cell atlas for normal and tumor samples from AML patients. **(A)** 8 cell types identified based on marker gene expression. **(B)** Compare the annotation results between normal and tumor tissues. **(C)** Show the proportions of eight cell types in AML patients and healthy controls. **(D)** Display whether there are differences in the proportions of 8 cell types between AML patients and healthy controls using box plots. **(E)** Show the expression of each MitoScore signature gene in the 8 cell types using a bubble chart. **(F)** Use the AUCell algorithm to score the activity of MitoScore signature genes in each cell type. **(G)** Display the scoring results using a UMAP plot. **(H)** Display whether there are differences in the activity scores of MitoScore signature genes between AML patients and healthy controls for the 8 cell types using box plots.

Using the “single” package, the CellMarker database, and reference (25), we identified marker genes to annotate different cell clusters. This process resulted in the identification of seven cell clusters: B Cell, Erythrocyte, Granulocyte, Macrophage, Megakaryocyte, Natural Killer Cell, and T Cell. A small fraction of cells that could not be recognized were annotated as unrecognizable. Since leukemia cells do not contain neurons, they were not considered core cell populations for subsequent analysis (**Figure 4A**). We further compared the annotation results and the proportions of these seven cell types between the healthy group and the AML group (**Figures 4B, C**). Box plots were used to illustrate the differences in the proportions of these seven cell types between the healthy and AML groups. The analysis revealed that the numbers of NK cells and T cells were significantly higher in the healthy group compared to the AML group (**Figure 4D**). The expression of 31 key mitochondrial genes in each cell type was visualized using bubble plots, which showed that DGUOK, FDPS, FTH1, HIGD2A, NDUFC1, NME3, UQCR10, and UQCR11 had higher expression levels in T cells (**Figure 4E**). Additionally, based on the gene set composed of these 31 key mitochondrial genes, we used the AUCell algorithm to identify subpopulations with active mitochondrial function. The identified active subpopulations were utilized to study the expression patterns of mitochondrial response genes at the single-cell level. The results indicated that T cells were among the active cell populations, and there was a difference in the activity scores of T cells between the healthy and AML groups (**Figures 4F–H**). Based on a comprehensive consideration of both quantity and mitochondrial scores, we will continue to perform further analysis on T cells.

### 3.5 Further pseudotime and cell communication analysis of T cell subsets

**Figure 5:**
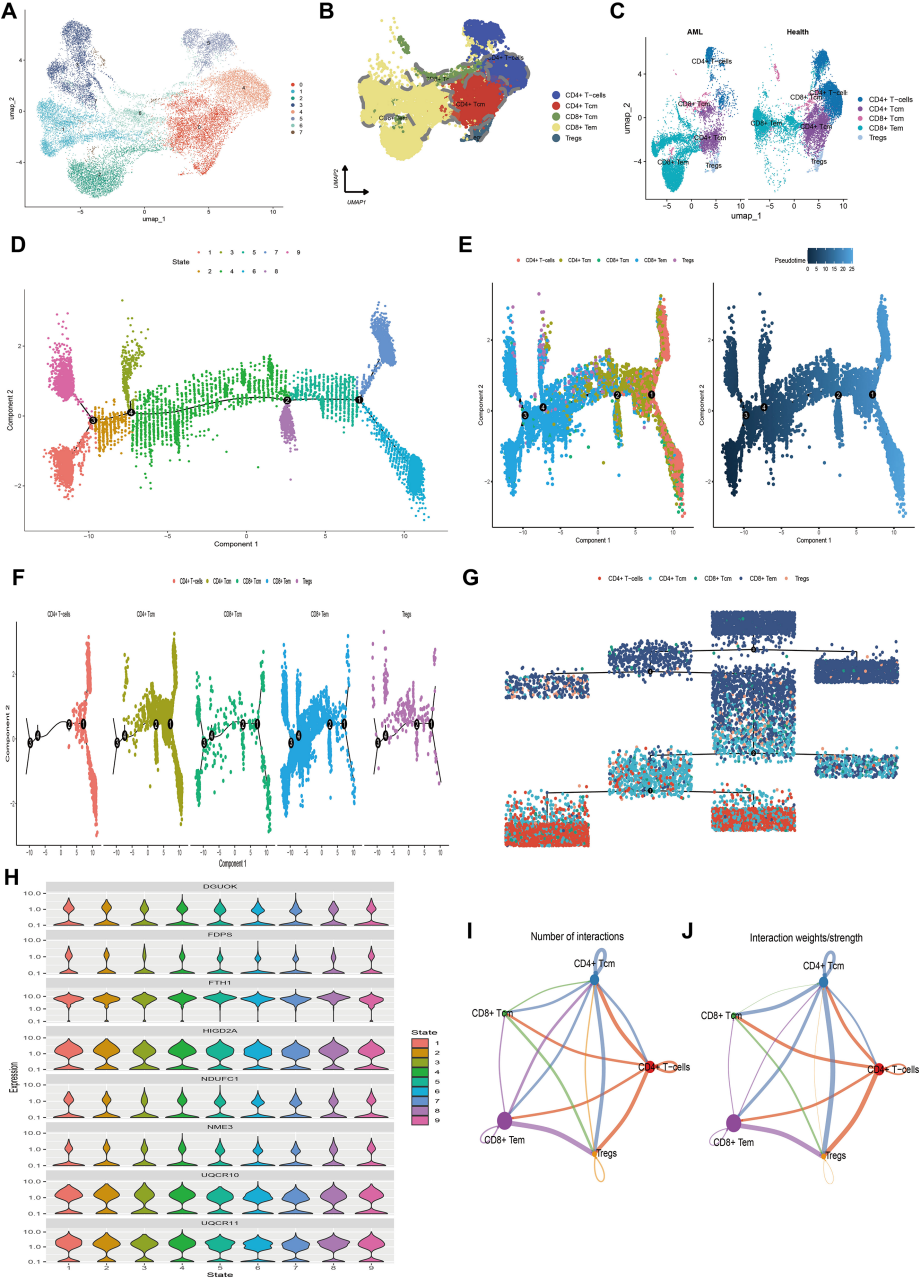
The single-cell transcriptional atlas of T cells and pseudo-time analysis and cell communication analysis of its five subtypes. **(A)** UMAP plot of T cells in scRNA-seq, clustered into 8 distinct clusters. **(B)** 5 cell types identified based on marker gene expression. **(C)** Compare the annotation results between normal and tumor tissues. **(D)** Trajectory plot showcasing the different stages of T cell differentiation along a pseudo-time axis. **(E)** Independent visualization of T cell subtypes at different stages along the pseudotime axis and trajectory plots depicting the developmental time course of T cells. **(F)** Trajectory plot of T cell subtypes indicating their differentiation patterns along a pseudo-time axis. **(G)** Independent visualization of T cell subtypes at different stages based on a dendrogram. **(H)** Violin plots of the expression levels of 8 MitoScore signature genes that are highly expressed in T cells, indicating their expression patterns along the pseudotime axis. **(I)** Number of interactions between 5 T cell subtypes. **(J)** Strength of interactions between 5 T cell subtypes.

To further characterize T cells, we isolated all T cells from the entire cell population. We performed dimensionality reduction and clustering analysis using the Uniform Manifold Approximation and Projection (UMAP) algorithm. This analysis classified the T cells into eight distinct clusters (**Figure 5A**). Based on the expression patterns of marker genes, we annotated these clusters into five T cell subpopulations: CD4+ T cells, central memory CD4+ T cells (CD4+ Tcm), central memory CD8+ T cells (CD8+ Tcm), effector memory CD8+ T cells (CD8+ Tem), and regulatory T cells (Tregs) (**Figure 5B**). We then compared the distribution and proportions of these subpopulations between healthy individuals and AML patients (**Figure 5C**).

To explore the differentiation trajectories of the annotated T cell subpopulations, we conducted pseudotime analysis using the Monocle 2 algorithm. This analysis revealed nine distinct differentiation states during T cell development (**Figure 5D**). The differentiation sequence was as follows: CD4+ T cells differentiate into Tregs and CD4+ Tcm, which subsequently give rise to CD8+ Tcm and CD8+ Tem. This indicates that CD4+ T cells, CD4+ Tcm, and Tregs represent early stages of cellular development, while CD8+ Tcm and CD8+ Tem correspond to terminal differentiation stages (**Figures 5E–G**). To visualize the expression dynamics of mitochondrial key genes during this process, we used violin plots to display the expression levels of eight highly expressed mitochondrial genes across the nine differentiation states (**Figure 5H**). Heatmaps were also generated to present clustering results based on pseudotime-associated genes, identifying differentially expressed genes (DEGs) along the pseudotime trajectory. These DEGs were categorized into three distinct subgroups (**Supplementary Figures S4A, B**). Collectively, these findings simplify the complex process of T cell differentiation, delineating key stages and molecular changes that define the transition from early to terminal stages of T cell development.

Furthermore, we inferred a cell-cell communication network to predict intercellular communications based on specific pathways and ligand-receptor interactions. A chord diagram illustrating the number of ligand-receptor pairs revealed that cellular communication was more frequent among CD4+ Tcm, CD4+ T cells, Tregs, and CD8+ Tem (**Figure 5I**). Notably, the interaction frequency and strength were higher between CD4+ Tcm and CD8+ Tem, CD4+ Tcm and Tregs, as well as CD4+ Tcm and CD4+ T cells, whereas CD8+ Tcm had relatively fewer interactions with other cell types (**Figure 5J**). We also detailed the communication scenarios of these five T cell subpopulations within the MIF signaling pathway, the CCL signaling pathway, and the IL-16 signaling pathway (**Supplementary Figure S5**). These results highlight the significant intercellular communication among T cell subpopulations, particularly between CD4+ Tcm and other types, which is crucial for immune coordination. This information could be essential for developing therapeutic strategies that target immune responses in AML.

### 3.6 Annotation of clinical characteristics for the MitoScore

To further investigate the relationship between MitoScore and clinical-pathological parameters and to validate its prognostic capability in AML, we conducted stratified analyses based on MitoScore about age, sex, cytogenetic risk, and FAB code. Our study revealed no significant differences in MitoScore between sexes (**Supplementary Figure S6B**). However, we observed significant associations between MitoScore and several other clinical parameters, including age, cyto_risk classification, and fab_code classification (**Supplementary Figures S6A, C, D**). To evaluate the impact of MitoScore on overall survival across different clinical characteristics, we performed a K-M survival analysis for patients stratified by age (<65 years or ≥65 years), sex, cytoreduction risk classification, and FAB classification. In each subgroup, patients with a high MitoScore exhibited significantly worse overall survival compared to those with a low MitoScore (**Supplementary Figures S6E–M**). These findings underscore the robust predictive power of the MitoScore for prognosis across multiple AML subgroups independent of traditional clinical factors.

### 3.7 Analysis of potential biological mechanisms of MitoScore signature

**Figure 6:**
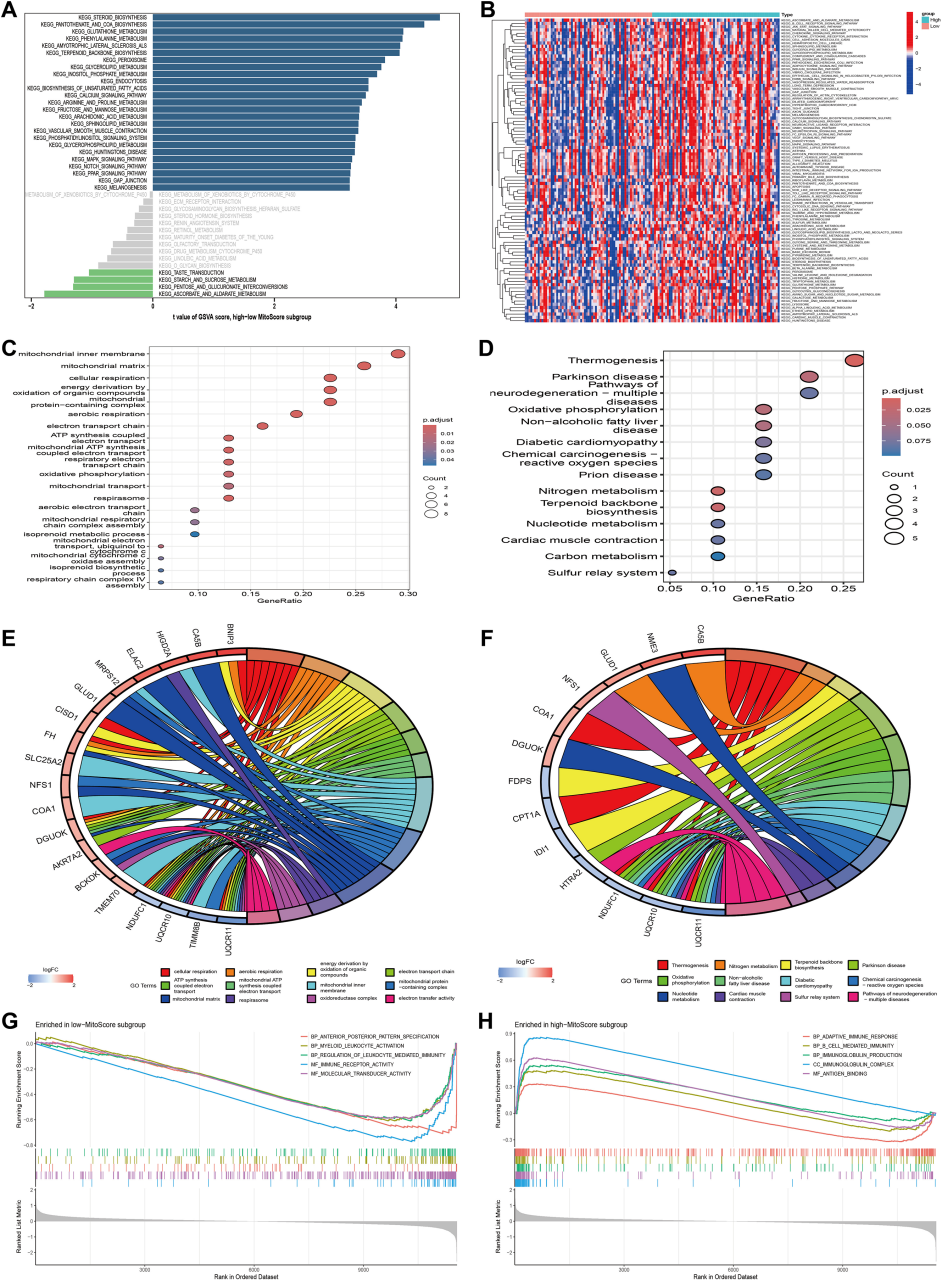
Functional enrichment analysis of MitoScore signature. **(A, B)** GSVA enriched pathways in the high- and low-MitoScore subgroup. **(C, E)** The GO enrichment analysis of the 31 key MitoScore signature genes. **(D, F)** The KEGG enrichment analysis of the 31 key MitoScore signature genes. **(G)** The top 5 GSEA enriched pathways in the low- MitoScore subgroup. **(F)** The top 5 GSEA enriched pathways in the low- MitoScore subgroup.

To gain deeper insights into the biological processes associated with the MitoScore signature, we performed comprehensive enrichment analyses. GSVA revealed that the MitoScore is significantly associated with cellular metabolism, particularly pathways related to carbohydrate metabolism, amino acid metabolism, and lipid metabolism (**Figures 6A, B**). Subsequently, we conducted an in-depth analysis of the 31 key mitochondrial genes using both Gene Ontology (GO) and Kyoto Encyclopedia of Genes and Genomes (KEGG) pathway analyses. GO analysis identified significant enrichment in biological processes, including mitochondrial transport, oxidative phosphorylation, the respiratory electron transport chain, mitochondrial ATP synthesis, and other pathways integral to mitochondrial and cellular respiration (**Figures 6C, E**). KEGG analysis highlighted enriched pathways, including terpenoid backbone biosynthesis, nitrogen metabolism, oxidative phosphorylation, thermogenesis, and various metabolic processes and disease mechanisms (**Figures 6D, F**).

To further elucidate the potential pathways associated with MitoScore, we employed Gene Set Enrichment Analysis (GSEA). In patients with lower MitoScores, we observed enrichment in biological processes and molecular functions related to anterior/posterior pattern specification, myeloid leukocyte activation, leukocyte-mediated immune regulation, immune receptor activity, and molecular transduction activity (**Figure 6G**). Conversely, patients with higher MitoScores showed enrichment in pathways associated with adaptive immune response, B-cell-mediated immunity, immunoglobulin production, immunoglobulin complexes, and antigen-binding (**Figure 6H**). These findings indicate that the MitoScore model is primarily enriched in pathways related to cellular metabolism, signal transduction, and immune responses. The comprehensive investigation of these pathways provides valuable insights into the specific biological mechanisms underlying the observed differences between MitoScore subgroups. This analysis highlights the potential significance of the 31 key mitochondrial genes in various KEGG pathways, underscoring their role in AML pathogenesis and prognosis.

### 3.8 Mutation landscape analysis of MitoScore signature

**Figure 7:**
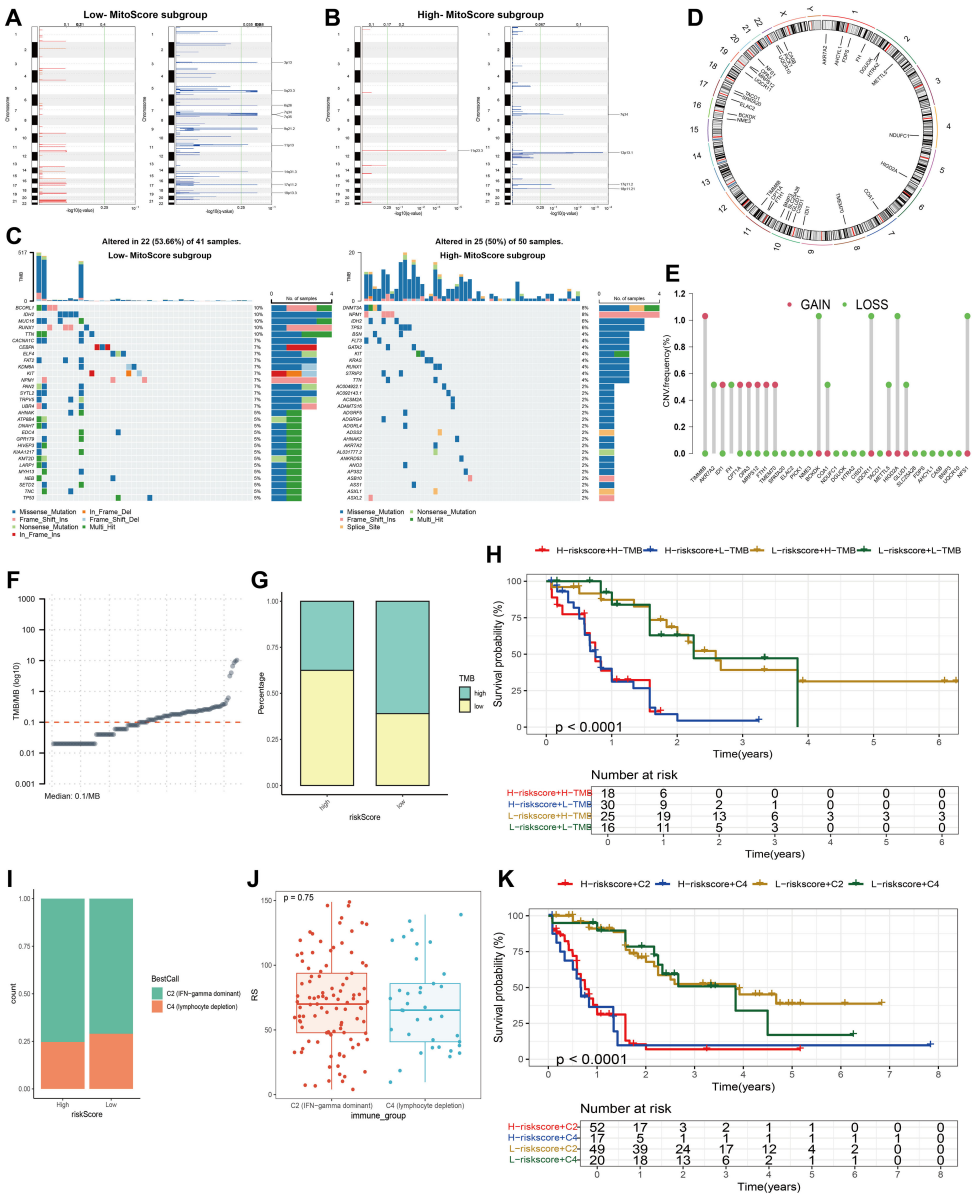
Mutation landscape analysis of MitoScore signature genes. **(A, B)** Recurrent regions of copy number amplification and deletion in the low and high- MitoScore subgroup. **(C)** Top 30 mutated genes were illustrated in the low- (left)and high- (right) MitoScore signature. **(D)** The chromosomal location of each MitoScore signature genes. **(E)** The CNV frequency of each MitoScore signature genes. **(F)** TMB/MB (log10) Demonstration for Each Sample. **(G)** Proportions of High TMB and Low TMB in the high- and low-MitoScore subgroup. **(H)** MitoScore and TMB-categorized OS KM curves. **(I)** Proportions of Two Cancer Immune Subtypes in the high- and low-MitoScore subgroup. **(J)** Box Plot Demonstrates the Differences in MitoScore Between Two Cancer Immune Subtypes. **(K)** OS KM Curves Based on MitoScore and Two Cancer Immune Subtypes.

To elucidate the genetic landscape associated with different MitoScore profiles, we utilized the Maftools software package to investigate the distribution of somatic variants in AML patients. We observed distinct driver mutation patterns between the two MitoScore subgroups. Additionally, using GISTIC2.0, we identified the frequency of recurrent copy number alterations (CNAs) in both the high and low MitoScore groups. The study found that the low MitoScore group exhibited a higher frequency of recurrent copy number alterations (CNAs) compared to the high MitoScore group (**Figures 7A, B**). Specifically, in the low MitoScore group, the genes with higher mutation frequencies were BCORL1, IDH2, MUC16, and RUNX1. In contrast, in the high MitoScore group, DNMT3A, NPM1, IDH2, and TP53 exhibited higher mutation frequencies (**Figure 7C**). Furthermore, we illustrated the chromosomal locations of the 31 key mitochondrial genes (**Figure 7D**). Copy number variations (CNVs) play a crucial role in the development and progression of cancer. In our model, the genes with the highest CNV frequencies included TIMM8B, BCKDK, UQCR11, HIGD2A, and NFS1 (**Figure 7E**). Considering the significant role of TMB in determining individual responses to immunotherapy, we examined its relationship with MitoScore. The results indicated that TMB levels were generally consistent between the high and low MitoScore subgroups (**Figures 7F, G**). Notably, although tumor mutation burden (TMB) itself was not significantly associated with prognosis in our cohort, patients in the high MitoScore subgroup had considerably worse prognoses compared to those in the low MitoScore subgroup independently of TMB levels (**Figure 7H**). This suggests that mitochondrial gene dysregulation may drive poor outcomes through mechanisms distinct from global mutational load. Our study consistently emphasizes the importance of the 31 key MitoScore genes in AML. We comprehensively evaluated the diverse roles and characteristics of these genes across various cancers (**Supplementary Figures S7**, **S8**, **S9**).

### 3.9 MitoScore was associated with immune characterization in AML

Using ssGSEA clustering, we classified AML samples into two immune phenotypes: C2 (IFN-gamma dominant) and C4 (lymphocyte depletion), and illustrated the proportions of these immune phenotypes within the high and low MitoScore subgroups (**Figure 7I**). However, no significant differences in MitoScore were observed between the two immune phenotypes (**Figure 7J**). Notably, while our analysis revealed no statistically significant difference in survival across molecular and immune subtypes, these subtypes exhibited marked immunological heterogeneity in terms of immune cell infiltration and pathway activation. Importantly, patients in the high MitoScore subgroup had significantly worse prognoses compared to those in the low MitoScore subgroup across all immune phenotypes (**Figure 7K**). This suggests that mitochondrial dysfunction drives adverse outcomes through mechanisms independent of tumor immune microenvironment characteristics. Additionally, we analyzed the expression levels of the 31 key MitoScore genes in these two immune phenotypes. Remarkably, the expression levels of AKR7A2, CISD1, GLUD1, HTRA2, MRPS12, NME3, and UQCR11 showed significant differences between the two immune phenotypes (**Supplementary Figure S10**).

**Figure 8:**
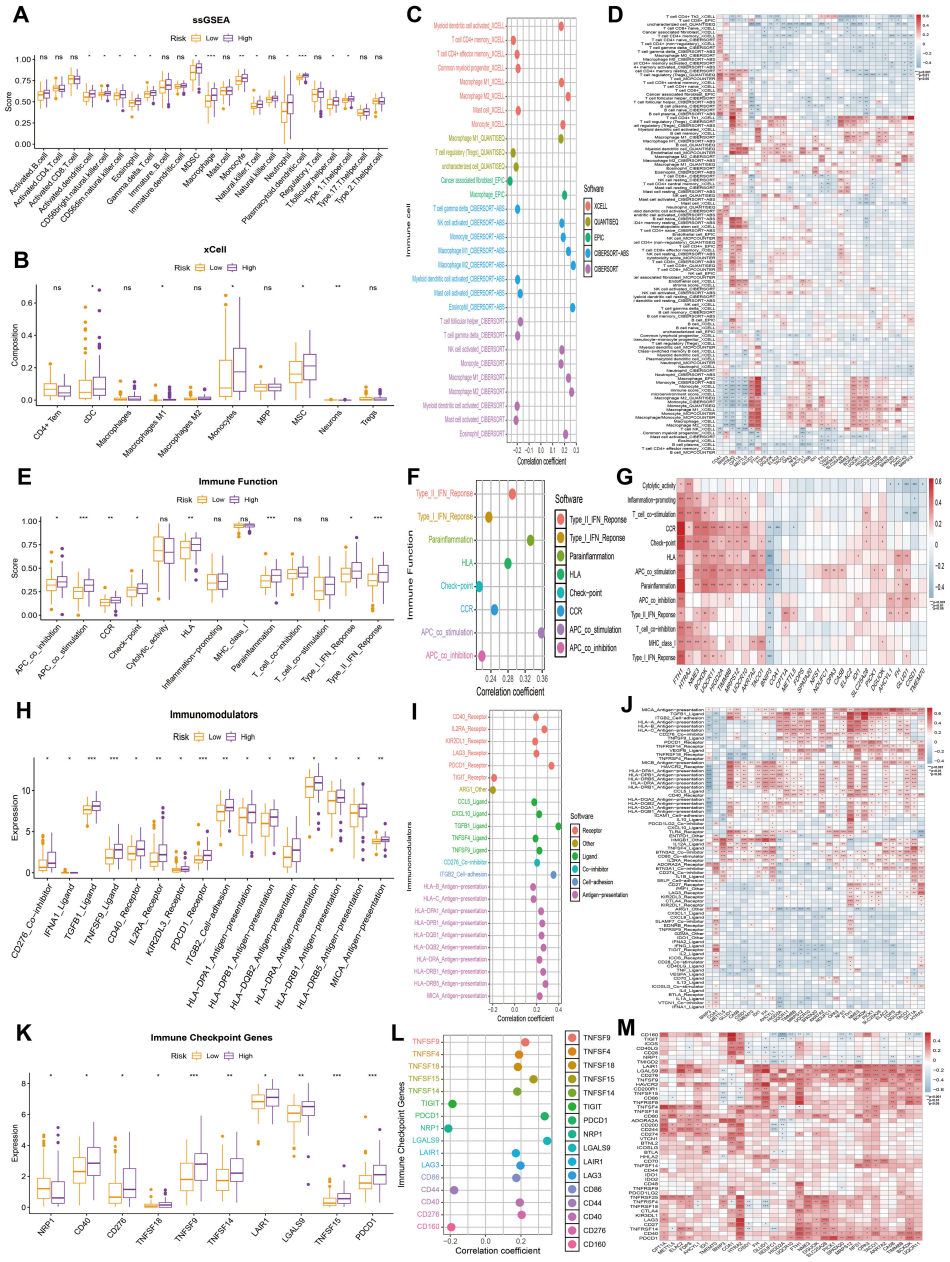
MitoScore and Immune-Related Analyses. **(A, B)** Display the differences in immune infiltration cells between high- and low-MitoScore subgroups using ssGSEA and xCell algorithms in a box plot. **(C)** Evaluate the correlation between immune infiltration cells and MitoScore. **(D)** Revealed a heatmap revealing the correlation between each MitoScore signature gene expression and immune infiltration cells. **(E)** Display the differences in immune function between high- and low-MitoScore subgroups. **(F)** Evaluating the correlation between immune function and MitoScore. **(G)** Revealed a heatmap showing the correlation between the expression of each MitoScore signature gene and immune function. **(H)** Display the differences in immunomodulators between high- and low- MitoScore subgroups. **(I)** Evaluate the correlation between immunomodulators and MitoScore. **(J)** Revealed a heatmap showing the correlation between the expression of each MitoScore signature gene and immunomodulators. **(K)** Display the differences in immune checkpoint genes between high- and low-MitoScore subgroups. **(L)** Evaluate the correlation between immune checkpoint genes and MitoScore. **(M)** Revealed a heatmap showing the correlation between the expression of each MitoScore signature gene and immune checkpoint genes. *, **, *** represent p < 0.05, p < 0.01, and p < 0.001, respectively. "ns" means not significant (p >= 0.05).

Considering the critical role of immune infiltration in tumorigenesis, we first evaluated the differences in immune cell subpopulations between the high MitoScore and low MitoScore subgroups. Specifically, the high MitoScore subgroup exhibited higher proportions of macrophages, monocytes, and plasmacytoid dendritic cells compared to the low MitoScore subgroup (**Figures 8A–C**). Next, we investigated the differences in immune function between the high MitoScore and low MitoScore subgroups. Our analysis revealed that the high MitoScore subgroup demonstrated stronger immune functions. Specifically, the high MitoScore subgroup showed higher scores for antigen-presenting cell (APC) co-stimulation, parainflammation, and Type II interferon (IFN) response compared to the low MitoScore subgroup (**Figures 8E, F**).

Subsequently, we investigated the relationship between the high MitoScore and low MitoScore subgroups and their association with immune regulators. Specifically, the high MitoScore subgroup exhibited higher expression levels of immune regulators, including TGFB1 ligand, TNFSF9 ligand, and PDCD1 receptor, compared to the low MitoScore subgroup (**Figures 8H, I**). We also examined the relationship between the high MitoScore and low MitoScore subgroups and immune checkpoint genes. The high MitoScore subgroup showed higher expression levels of immune checkpoint genes, including TNFSF9, TNFSF15, and PDCD1, compared to the low MitoScore subgroup (**Figures 8K, L**). Furthermore, we used heatmaps to provide a detailed visualization of the distribution of the 31 key mitochondrial genes across immune cells, immune functions, immune regulators, and immune checkpoint genes, offering valuable insights for future research (**Figures 8D, G, J, M**).

### 3.10 Drug sensitivity analysis for MitoScore signature

**Figure 9:**
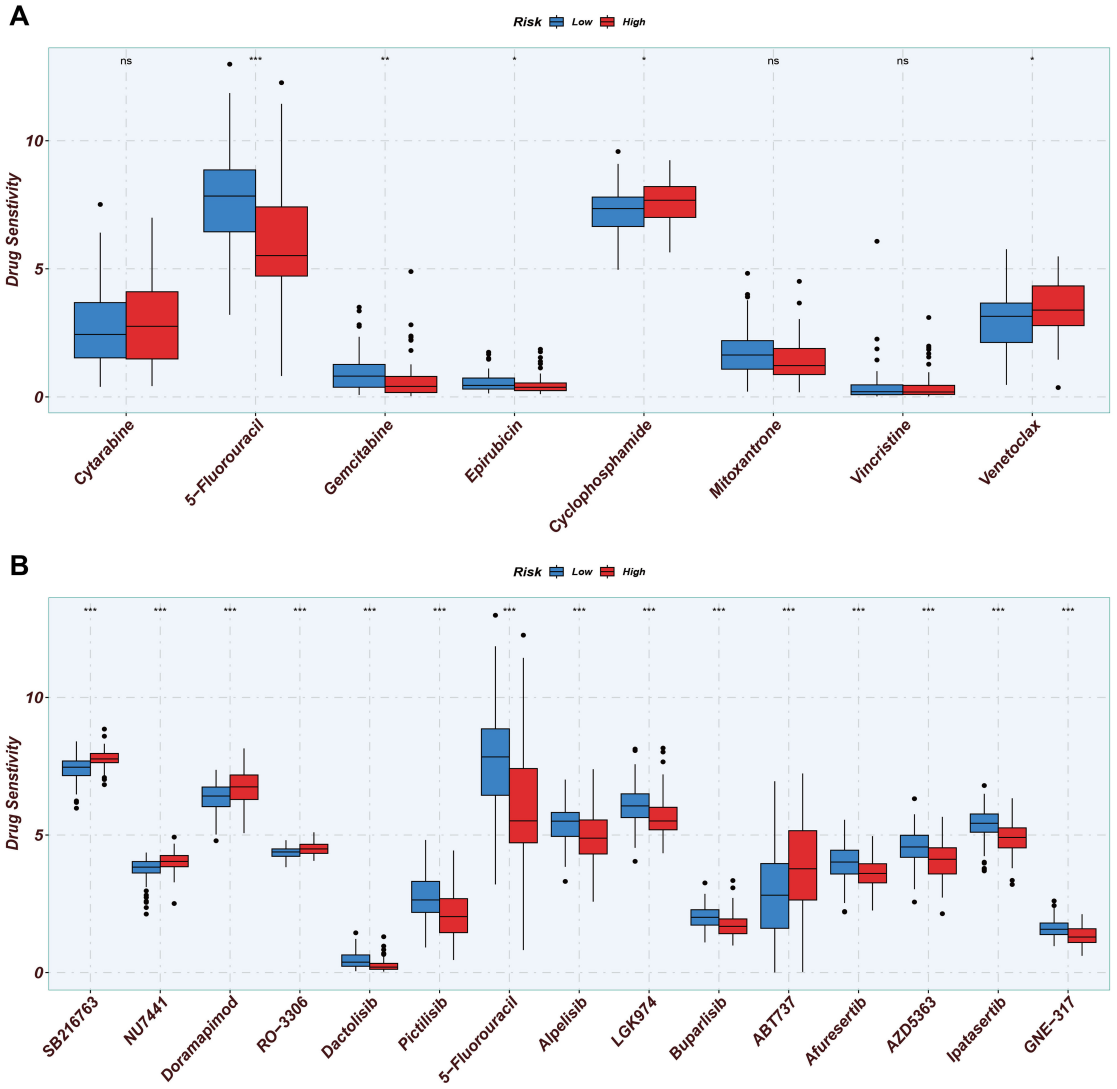
Drug sensitivity analysis of two MitoScore subgroups. **(A)** Display the drug sensitivity of current clinical AML treatments in high- and low-MitoScore subgroups. **(B)** Display the drug sensitivity of drugs not yet widely used in high- and low-MitoScore subgroups. *, **, *** represent p < 0.05, p < 0.01, and p < 0.001, respectively. "ns" means not significant (p >= 0.05).

From the Genomics of Drug Sensitivity in Cancer (GDSC) database, we identified significant differences in drug sensitivity between the high MitoScore and low MitoScore subgroups. We specifically examined eight drugs commonly used in the clinical treatment of AML patients and found that 5-fluorouracil, Gemcitabine, and Epirubicin were more sensitive in the low MitoScore subgroup. In contrast, Cyclophosphamide and Venetoclax were more sensitive in the high MitoScore subgroup (**Figure 9A**). Furthermore, we highlighted 15 drugs from the GDSC database that exhibited highly significant differences in sensitivity between the high MitoScore and low MitoScore subgroups. Drugs such as SB216763, NU7441, Doramapimod, RO-3306, and ABT737 showed greater sensitivity in the high MitoScore subgroup. At the same time, Dactolisib, Pictilisib, Alpelisib, LGK974, Buparlisib, Afuresertib, AZD5363, Ipatasertib, and GNE-317 were more sensitive in the low MitoScore subgroup (**Figure 9B**). In summary, these findings suggest that these drugs may represent promising therapeutic options for AML, with their effectiveness potentially guided by MitoScore status.

## 4 Discussion

To date, extensive research has elucidated the role of mitochondria in tumorigenesis, proliferation, and metastasis, primarily through their involvement in bioenergetics, signaling pathways, and biosynthesis (14, 15). The activation of oncogenes, inactivation of tumor suppressor genes, and accumulation of tumor-induced mutations in tricarboxylate circulating enzymes contribute to the production of excessive mitochondrial reactive oxygen species (ROS), which promote the development of tumor cells within the hypoxic microenvironment (35). Tumor cells exhibit elevated levels of antioxidant proteins that mitigate cell death induced by ROS-mediated alterations in mitochondrial permeability (36). Furthermore, the sustained accumulation of metabolites produced by mutant mitochondrial enzymes supports tumor cell proliferation (37). Changes in mitochondrial metabolic status influence various signaling pathways, enhance metabolic reprogramming, and facilitate tumor metastasis. Consequently, numerous anti-tumor agents have been developed to target specific vulnerabilities in mitochondrial metabolism. However, due to the adaptive and compensatory mechanisms of tumor cells and the off-target effects of mitochondrial-targeted drugs, the efficacy of single-target therapies is often limited (38). This raises the question of whether a gene set comprising multiple mitochondrial functional genes can be utilized to develop an AML risk model that predicts treatment outcomes and informs the selection of therapeutic regimens.

To our knowledge, this study represents the first comprehensive investigation of mitochondrial function in AML, identifying 31 key mitochondrial genes and constructing a MitoScore signature utilizing a combination of 148 algorithms derived from 14 machine-learning methods. In contrast to existing models, our signature was developed by integrating multiple datasets and machine learning approaches, and its robustness was validated using external datasets. Furthermore, we established a MitoScore-based nomogram designed to enhance the accuracy of clinical prognosis predictions in AML patients. This nomogram incorporates additional variables, including age, sex, cytogenetic risk, and French-American-British (FAB) classification. The nomogram demonstrated an AUC exceeding 0.8 for predicting outcomes at 1, 3, and 5 years, indicating strong predictive power and stability.

In addition, we obtained 39,783 cells in the scRNA-seq map and identified seven types of core cells: granulocytes, erythrocytes, megakaryocytes, macrophages, B cells, natural killer cells, and T cells. Compared with healthy controls, the number and mitochondrial function scores of T cells were reduced in AML patients, suggesting that AML may inhibit mitochondrial function by affecting mitochondrial nucleotide synthesis, enzyme activity and respiratory chain complex of T cells. To further understand the influence of mitochondrial function on T cells, we divided T cells into five subgroups and nine differentiation stages, analyzed the expression of key mitochondrial genes at different differentiation stages of various subgroups of cells, and found that different differentiation stages of T cells had specific expressions of key mitochondrial genes, which provided a possible method for simplifying the identification of T cell differentiation stages. In the communication network of T cell subsets in AML, CD4+ Tcm serves as a pivotal hub, facilitating interactions among various T cell subsets. Previous research has demonstrated that AML patients harboring DNMT3A mutations exhibit a reduction in naive CD8+ T cells and CD4+ effector memory T cells compared to controls without such mutations while concurrently showing an increase in CD4+ Tcm cells (39). Notably, an elevated level of CD4+ Tcm cells has been associated with poor prognosis in chronic lymphocytic leukemia (40).

Furthermore, the sustained proliferative capacity and robust effector function of CD4+ Tcm cells render them critical parameters for assessing the persistence and efficacy of chimeric antigen receptor (CAR) T cell therapy (41). Despite the remarkable success of CAR T therapies in treating B-cell lymphoblastic leukemia, B-cell lymphoma, and multiple myeloma, significant challenges remain in addressing acute myeloid leukemia (AML), primarily due to the absence of stable target antigens (42). The extent to which the findings of this study can serve as predictors of CAR T therapy efficacy in AML patients, or whether mitochondrial functional genes expressed explicitly by CD4+ Tcm cells may represent viable targets for CAR T therapy, necessitates further investigation supported by subsequent experiments and clinical data.

We categorized all samples into low-risk and high-risk groups based on the MitoScore signature. We then evaluated these two groups in terms of clinical features, biological mechanisms, copy number alterations, tumor mutational burden, immune infiltration, immune functions, and immune checkpoint gene expression. Our findings indicated that, compared to the low-risk group, the high-risk group exhibited a poorer survival prognosis, greater enrichment in immune response-related biological processes and molecular functional pathways, a higher frequency of mutations with adverse outcomes, increased infiltration of immune cells and immune function, and heightened expression of genes at immune checkpoints.

Finally, we screened effective drugs and potential compounds for patients with AML from the GDSC database, guided by the MitoScore signature. Among the commonly used therapies for AML, the high-risk group demonstrated greater sensitivity to cyclophosphamide and venetoclax, whereas the low-risk group responded more favorably to 5-fluorouracil, gemcitabine, and epirubicin. Furthermore, we identified 15 compounds with therapeutic efficacy against AML, which exhibited significant differences in sensitivity between the low-risk and high-risk groups.

This study lays a foundation for the stratified and precise treatment of patients with AML; however, it is not without limitations. First, the establishment of our model primarily relies on bulk RNA-seq data, which is relatively limited in scope. Future research should incorporate scRNA-seq data and conduct internal data analyses to enhance the predictive efficiency of the model. Additionally, the scarcity of data may contribute to inconsistencies observed between the analysis of CNVs in different groups and the findings of previous studies. Third, immune infiltration analyses may be confounded by technical batch effects and tumor heterogeneity—despite employing batch correction algorithms (e.g., ComBat) and using uniformly processed TCGA data. Inherent limitations of bulk RNA-seq deconvolution methods (CIBERSORT/ssGSEA) constrain the interpretation of the microenvironment. Finally, the regulatory mechanisms of the key mitochondrial genes that comprise the MitoScore signatures in AML remain unclear, and there is a lack of corresponding literature to support this understanding. These aspects warrant further verification and investigation through subsequent experiments utilizing three-dimensional models: cellular, animal, and clinical specimens.

Moreover, our study has three critical limitations: (1) the absence of external validation using independent clinical cohorts for the prognostic model; (2) insufficient experimental interrogation of key genes’ biological functions in AML progression; and (3) lack of qPCR validation for mitochondrial gene expression patterns, restricting confirmation to transcriptomic-level observations without multilevel biological corroboration. We acknowledge that future work must address the clinical applicability and biological relevance of our findings through validation with independent prospective clinical cohorts to confirm model generalizability, mechanistic experiments (e.g., knockdown/overexpression of hub genes in AML models) to elucidate regulatory roles, and integrated molecular validation combining qPCR, protein analysis, and functional assays to establish translational relevance.

## 5 Conclusion

By integrating mitochondrial genes with bulk RNA-seq data from AML and employing various machine-learning methods, we developed a novel prognostic model that effectively predicts patient survival outcomes. Moreover, the MitoScore facilitates the stratification of patients into low-risk and high-risk groups, with distinct gene mutation profiles, immune characteristics, and drug sensitivities observed between these groups. This study presents a reliable prognostic model for AML, opening new avenues for personalized treatment strategies in the future.

## Data Availability

The original contributions presented in the study are included in the article/**Supplementary Material**. Further inquiries can be directed to the corresponding author.

## Supplementary material

Supplementary Figure 1 The expression pattern of MitoScore signature genes in tumor and normal. A The box plot depicts the differences of each MitoScore signature gene between tumor and normal tissues. B The ridge plot illustrates the expression patterns of each MitoScore signature gene.

Supplementary Figure 2 The expression pattern and survival analysis of MitoScore signature genes in pan-cancer analysis. **(A)** Summarizes the expression difference between normal and cancer groups. **(B)** Summarizes the survival difference between high- and low-gene expression groups.

Supplementary Figure 3 The nomogram model was constructed based on Univariate and multivariate cox regression analyses. **(A)** Nomogram to predict 1-, 3-, and 5-year AML patient survival. **(B)** Nomogram calibration curves for 1-, 3-, and 5-year OS. **(C)** The ROC curves for the MitoScore signature are displayed. **(D)** The ROC curves of the MitoScore signature for 1, 3, and 5 years. **(E)** AUC analysis of each variable included in the nomogram model for 1 years. **(F)** AUC analysis of each variable included in the nomogram model for 3 years. **(G)** AUC analysis of each variable included in the nomogram model for 5 years. **(H)** DCA curves were compared over a period for patients with AML. **(I)** DCA curves were compared over a period of 1 year, 3 years, and 5 years for patients with AML.

Supplementary Figure 4
**(A)** Cluster heatmap based on pseudo-time series related genes. **(B)** DEGs identified along the pseudo-temporal trajectory are categorized into three distinct subgroups

Supplementary Figure 5 Cell-cell communication analysis. **(A–C)** Intercellular communication network diagram within the MIF signaling pathway. **(D)** Heatmap displaying the interaction patterns of ligand-receptor interactions in the MIF signaling pathway across different cell types. **(E)** Expression profiles of representative genes in the MIF signaling pathway across different cell types. **(F–H)** Intercellular communication network diagram within the CCL signaling pathway. **(I)** Heatmap displaying the interaction patterns of ligand-receptor interactions in the CCL signaling pathway across different cell types. **(J)** Expression profiles of representative genes in the CCL signaling pathway across different cell types. **(K–M)** Intercellular communication network diagram within the IL16 signaling pathway. **(N)** Heatmap displaying the interaction patterns of ligand-receptor interactions in the IL16 signaling pathway across different cell types. **(O)** Expression profiles of representative genes in the IL16 signaling pathway across different cell types.

Supplementary Figure 6 Annotation of clinical characteristics for the MitoScore. **(A–D)** The expression of MitoScore signature in different clinical subgroups. **(E–M)** Kaplan-Meier survival analyses of MitoScore signature in different strata of clinical characteristics.

Supplementary Figure 7 The CNV landscape of MitoScore signature genes in pan-cancer analysis. **(A)** Correlations between CNV and mRNA expression of each MitoScore signature gene in the pan cancers. **(B)** CNV percentage of each MitoScore signature gene in each cancer. **(C)** Summarizes the survival difference between CNV groups.

Supplementary Figure 8 The methylation landscape of MitoScore signature genes in pan-cancer analysis. **(A)** The methylation difference between tumor and normal samples in the pan cancers. **(B)** Correlations between methylation and mRNA expression of each MitoScore signature gene in the pan cancers. **(C)** Summarizes the survival difference between high and low methylation groups.

Supplementary Figure 9 The mutation landscape of MitoScore signature genes in pan-cancer analysis. **(A)** Copilot of the signature gene mutation frequency in pan cancers. **(B, C)** Copilot of the single-nucleotide variant in pan cancers. **(D)** Summarizes the survival difference between mutant and WT.

Supplementary Figure 10 Box plot portrays the dissimilarities in the cancer immunity subgroup between MitoScore signature genes.

Supplementary TABLE 1 All of the included Mitochondria-related genes.
