# Supplementary figures and images for: Integrated bioinformatic analysis and machine learning developed a prognostic model based on mitochondrial function for acute myeloid leukemia
Source: Front Immunol. 2025 Oct 23;16:1597633. doi: 10.3389/fimmu.2025.1597633 (PMC12588975; doi:10.3389/fimmu.2025.1597633)

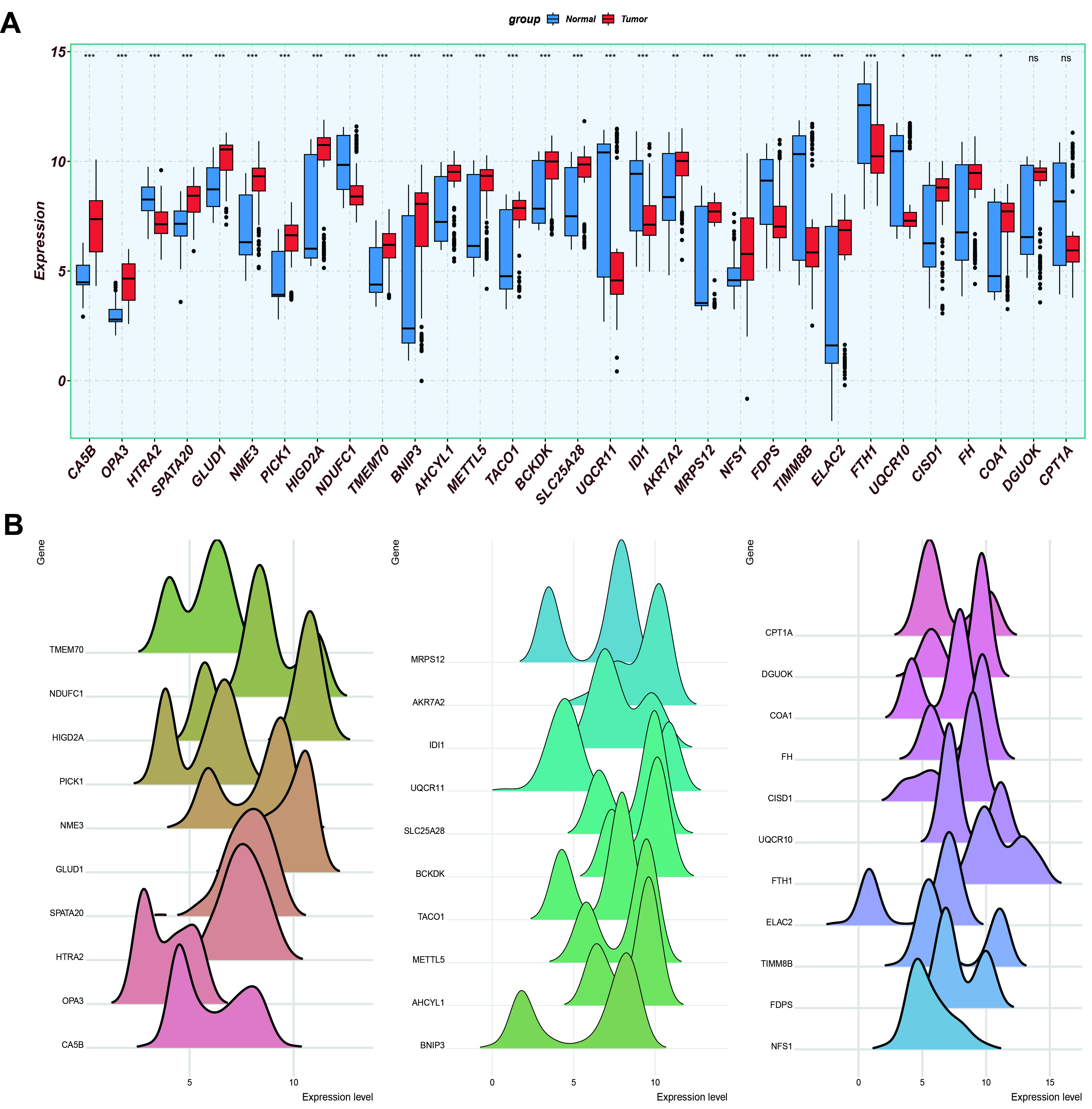

Supplement: Supplementary Figure 1 — The expression pattern of MitoScore signature genes in tumor and normal. A The box plot depicts the differences of each MitoScore signature gene between tumor and normal tissues. B The ridge plot illustrates the expression patterns of each MitoScore signature gene. [file Image1.jpeg]

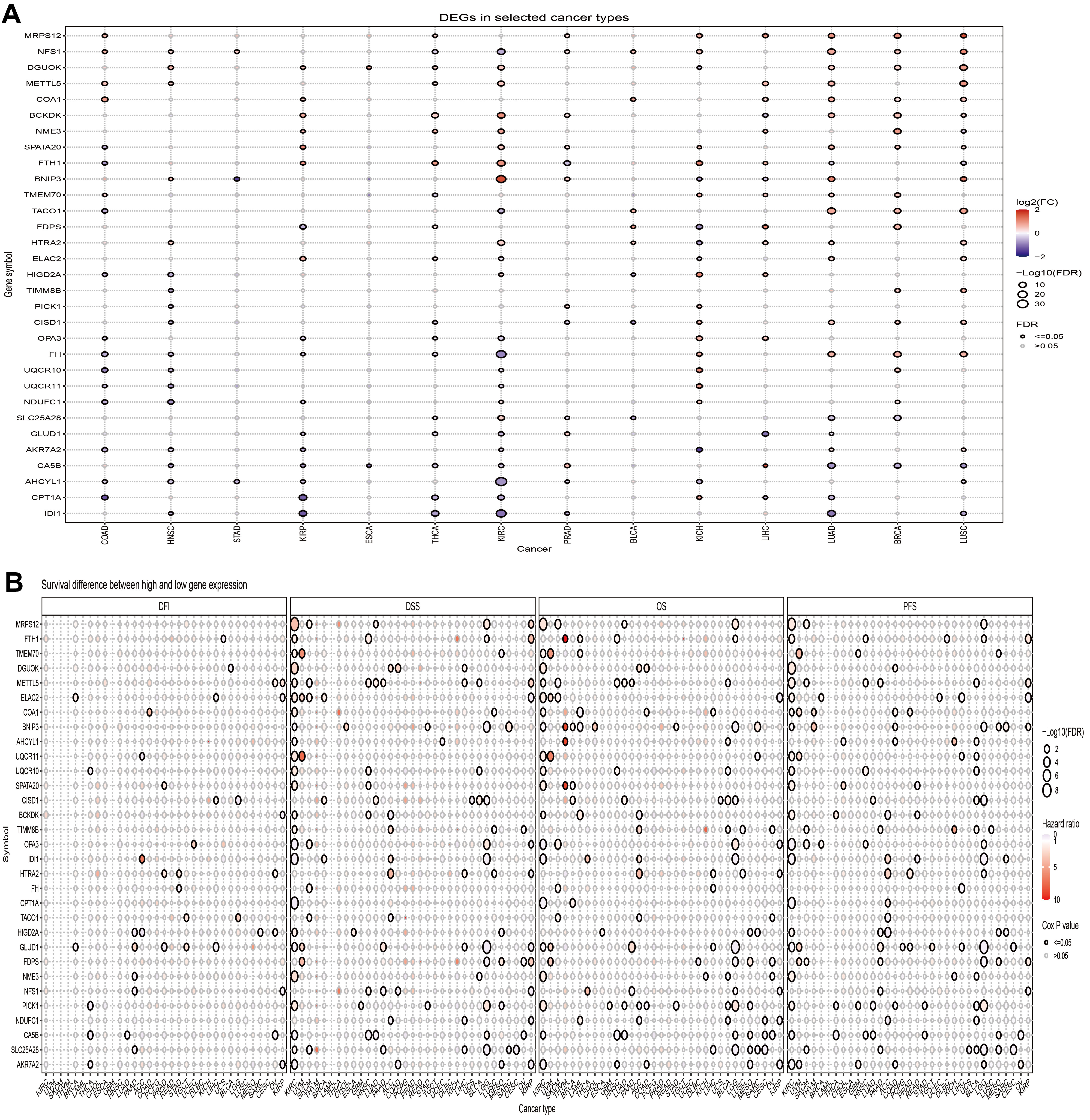

Supplement: Supplementary Figure 2 — The expression pattern and survival analysis of MitoScore signature genes in pan-cancer analysis. (A) Summarizes the expression difference between normal and cancer groups. (B) Summarizes the survival difference between high- and low-gene expression groups. [file Image2.jpeg]

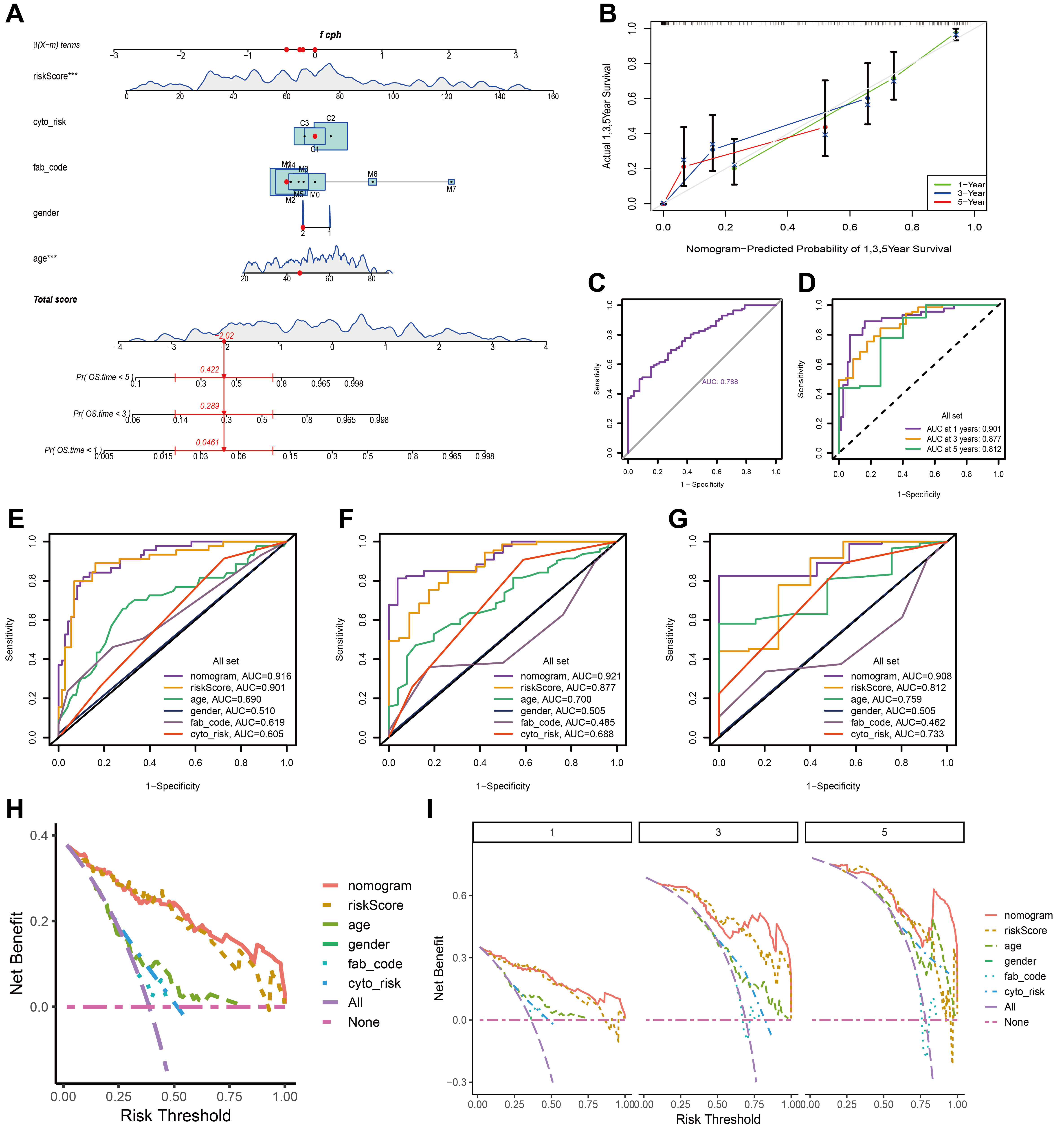

Supplement: Supplementary Figure 3 — The nomogram model was constructed based on Univariate and multivariate cox regression analyses. (A) Nomogram to predict 1-, 3-, and 5-year AML patient survival. (B) Nomogram calibration curves for 1-, 3-, and 5-year OS. (C) The ROC curves for the MitoScore signature are displayed. (D) The ROC curves of the MitoScore signature for 1, 3, and 5 years. (E) AUC analysis of each variable included in the nomogram model for 1 years. (F) AUC analysis of each variable included in the nomogram model for 3 years. (G) AUC analysis of each variable included in the nomogram model for 5 years. (H) DCA curves were compared over a period for patients with AML. (I) DCA curves were compared over a period of 1 year, 3 years, and 5 years for patients with AML. [file Image3.jpeg]

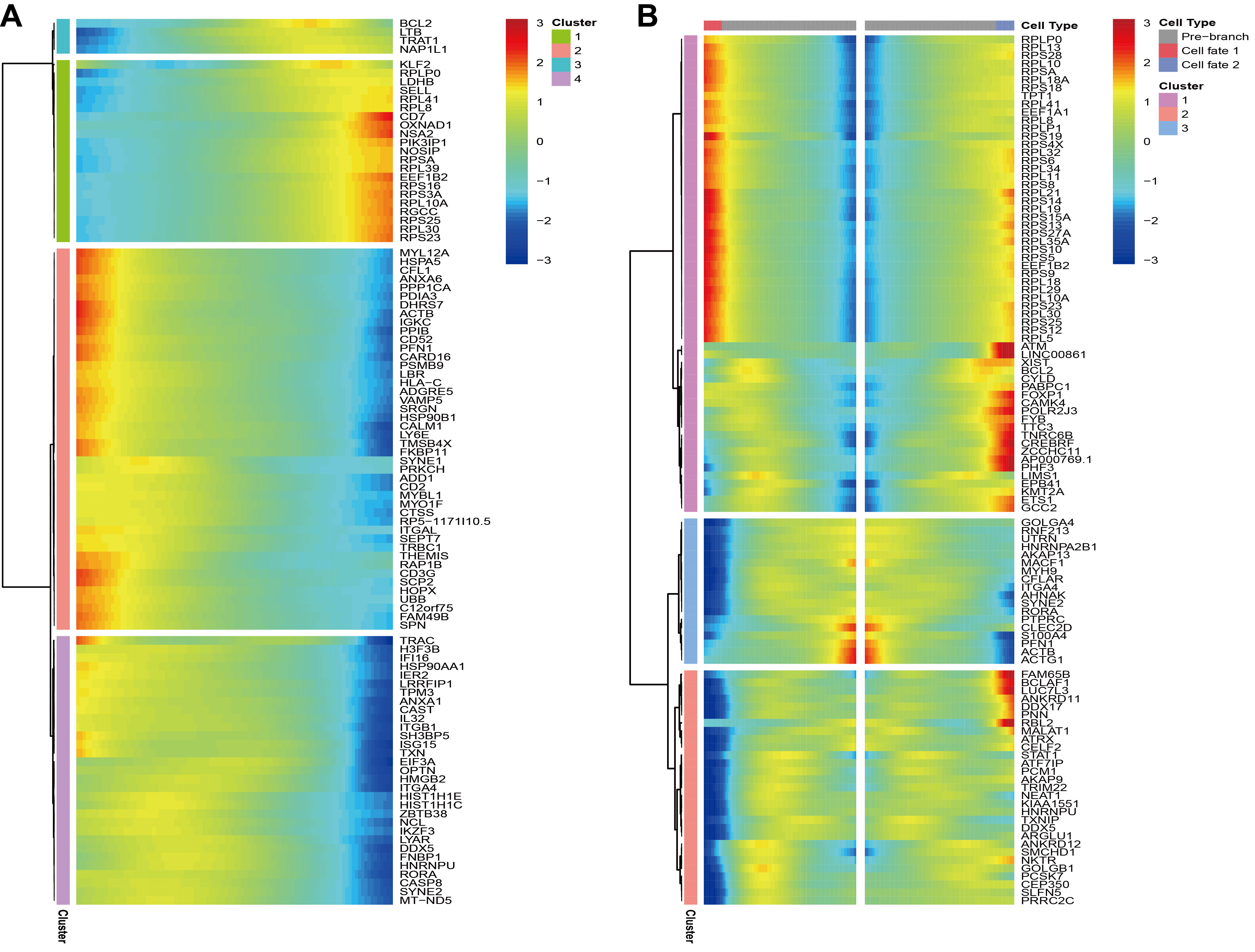

Supplement: Supplementary Figure 4 — (A) Cluster heatmap based on pseudo-time series related genes. (B) DEGs identified along the pseudo-temporal trajectory are categorized into three distinct subgroups [file Image4.jpeg]

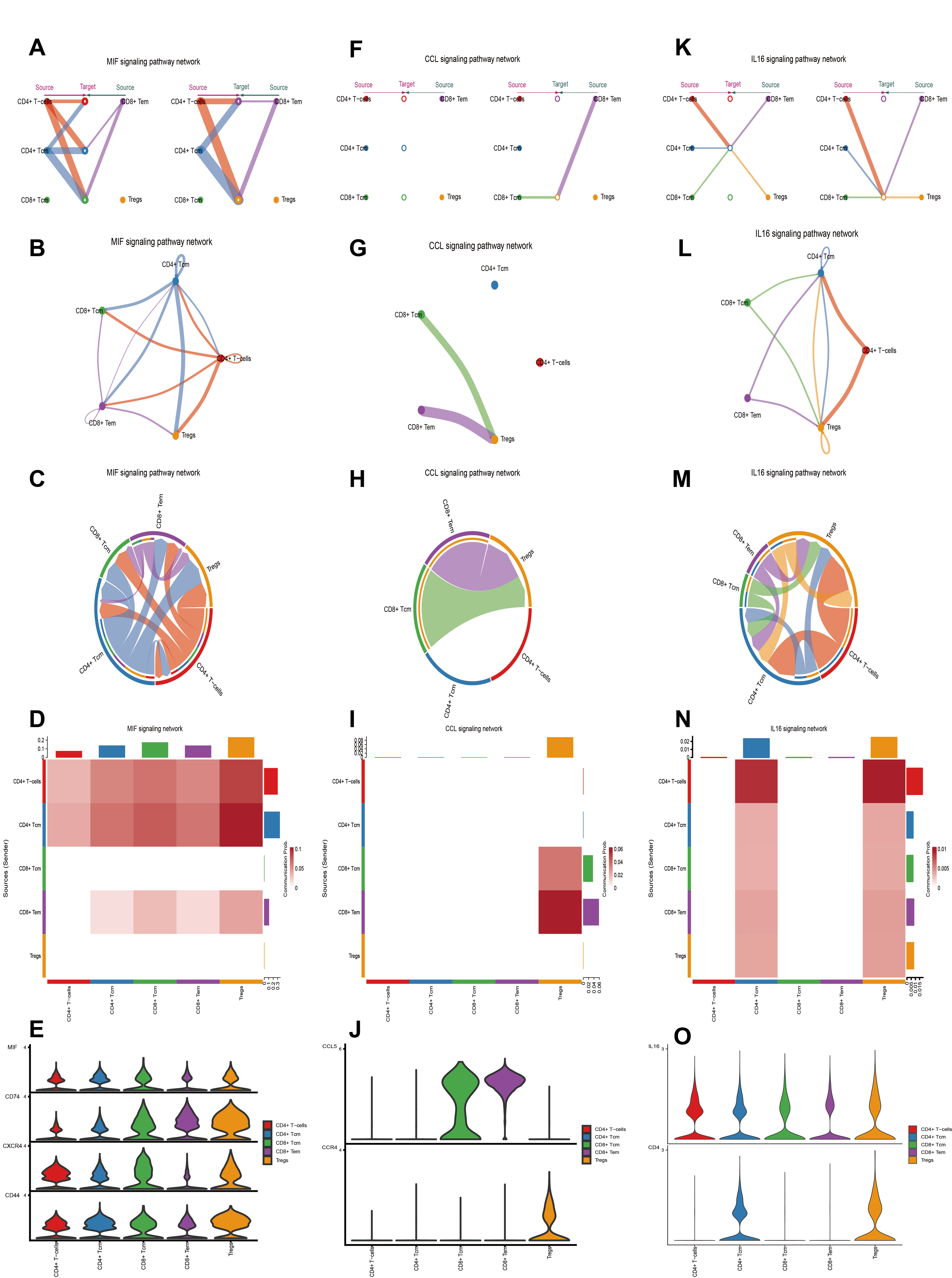

Supplement: Supplementary Figure 5 — Cell-cell communication analysis. (A–C) Intercellular communication network diagram within the MIF signaling pathway. (D) Heatmap displaying the interaction patterns of ligand-receptor interactions in the MIF signaling pathway across different cell types. (E) Expression profiles of representative genes in the MIF signaling pathway across different cell types. (F–H) Intercellular communication network diagram within the CCL signaling pathway. (I) Heatmap displaying the interaction patterns of ligand-receptor interactions in the CCL signaling pathway across different cell types. (J) Expression profiles of representative genes in the CCL signaling pathway across different cell types. (K–M) Intercellular communication network diagram within the IL16 signaling pathway. (N) Heatmap displaying the interaction patterns of ligand-receptor interactions in the IL16 signaling pathway across different cell types. (O) Expression profiles of representative genes in the IL16 signaling pathway across different cell types. [file Image5.jpeg]

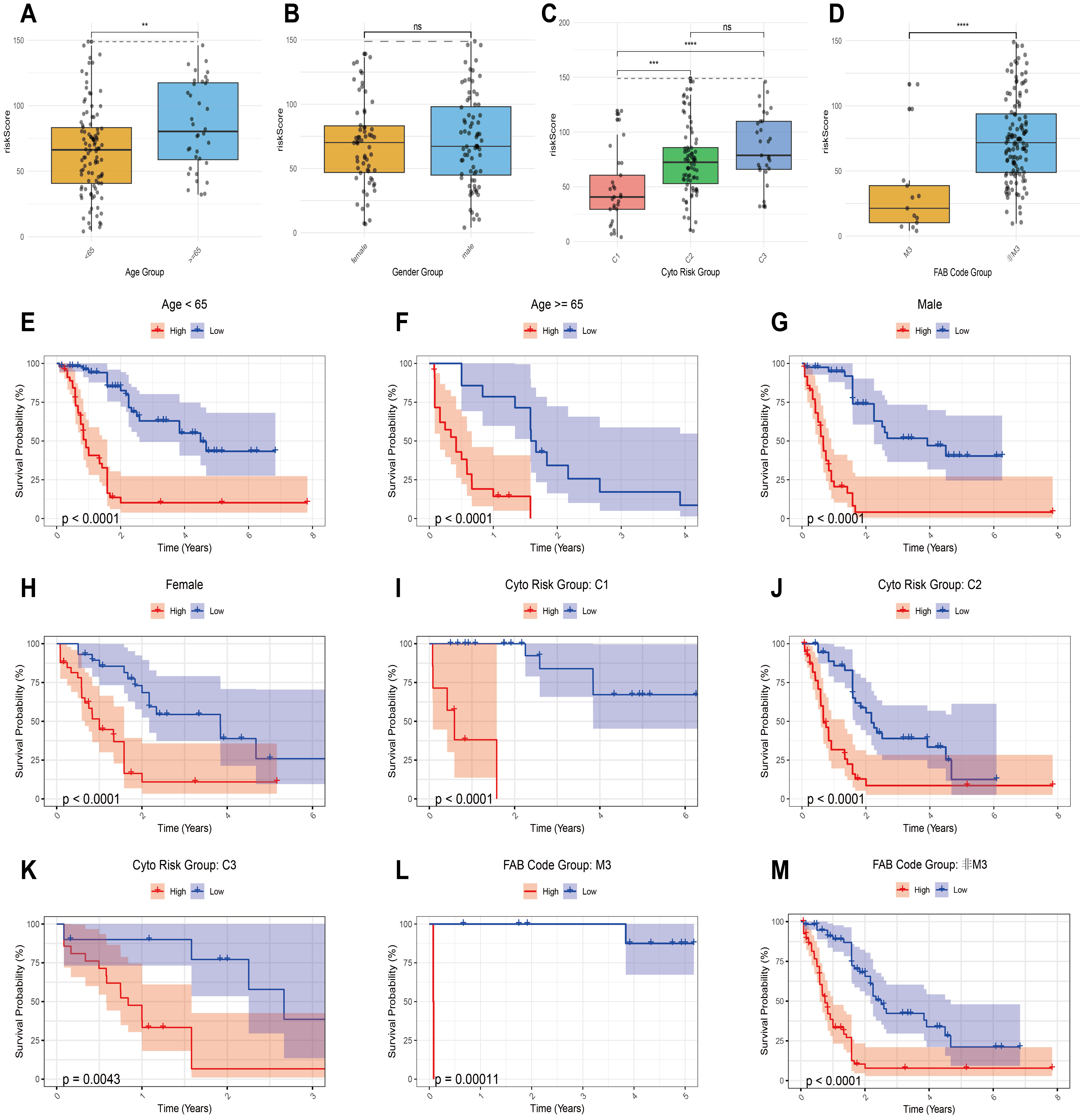

Supplement: Supplementary Figure 6 — Annotation of clinical characteristics for the MitoScore. (A–D) The expression of MitoScore signature in different clinical subgroups. (E–M) Kaplan-Meier survival analyses of MitoScore signature in different strata of clinical characteristics. [file Image6.jpeg]

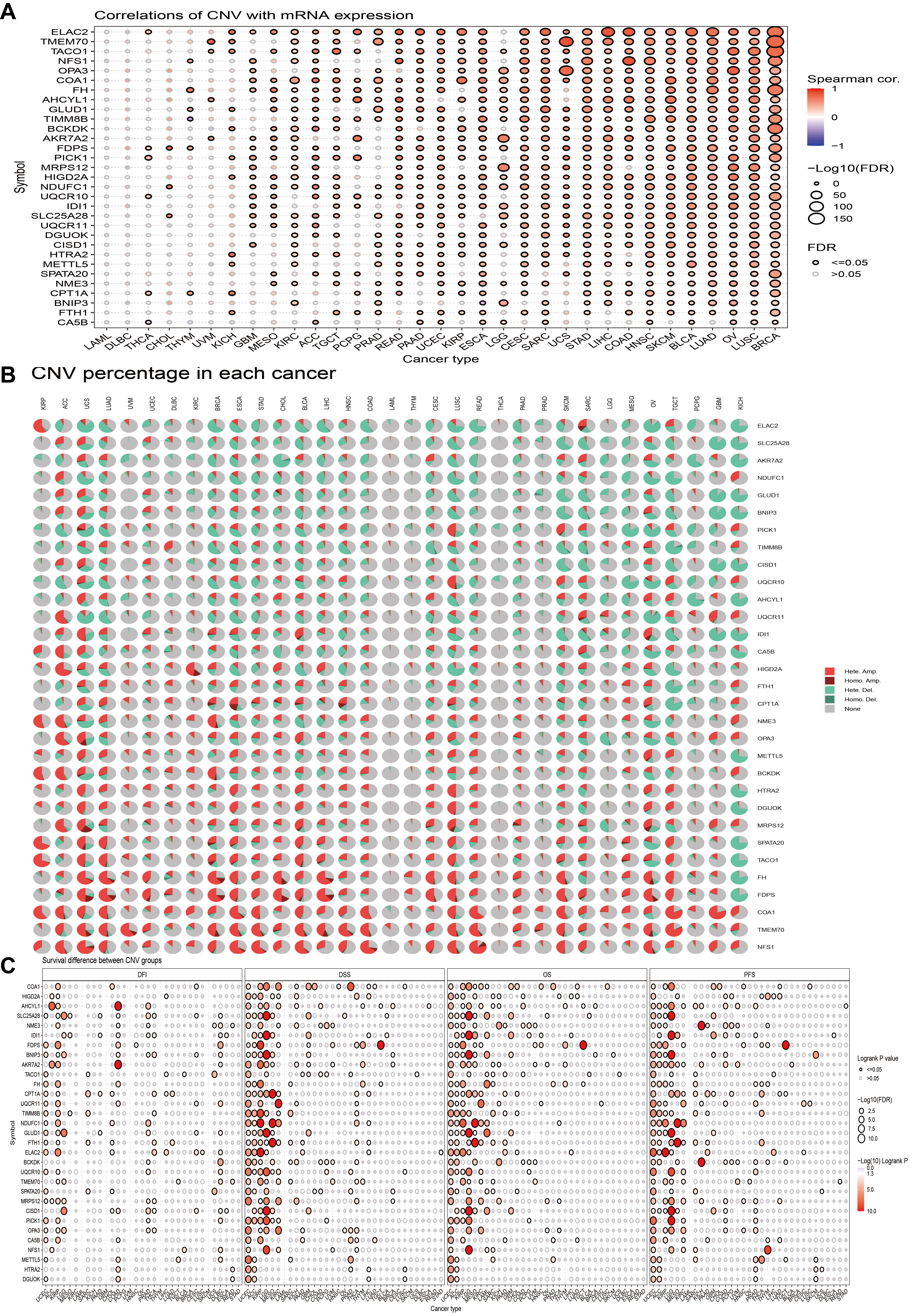

Supplement: Supplementary Figure 7 — The CNV landscape of MitoScore signature genes in pan-cancer analysis. (A) Correlations between CNV and mRNA expression of each MitoScore signature gene in the pan cancers. (B) CNV percentage of each MitoScore signature gene in each cancer. (C) Summarizes the survival difference between CNV groups. [file Image7.jpeg]

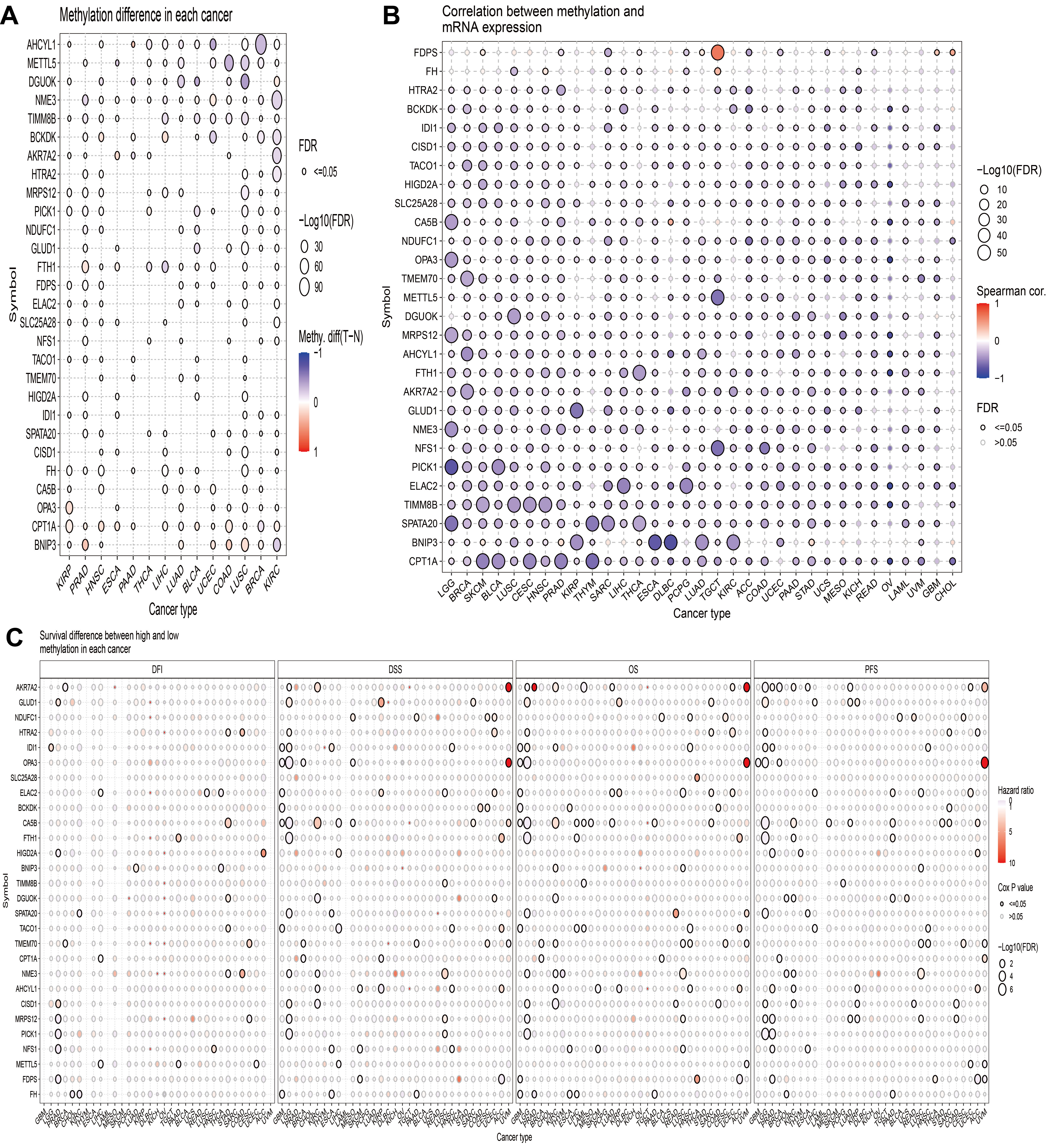

Supplement: Supplementary Figure 8 — The methylation landscape of MitoScore signature genes in pan-cancer analysis. (A) The methylation difference between tumor and normal samples in the pan cancers. (B) Correlations between methylation and mRNA expression of each MitoScore signature gene in the pan cancers. (C) Summarizes the survival difference between high and low methylation groups. [file Image8.jpeg]

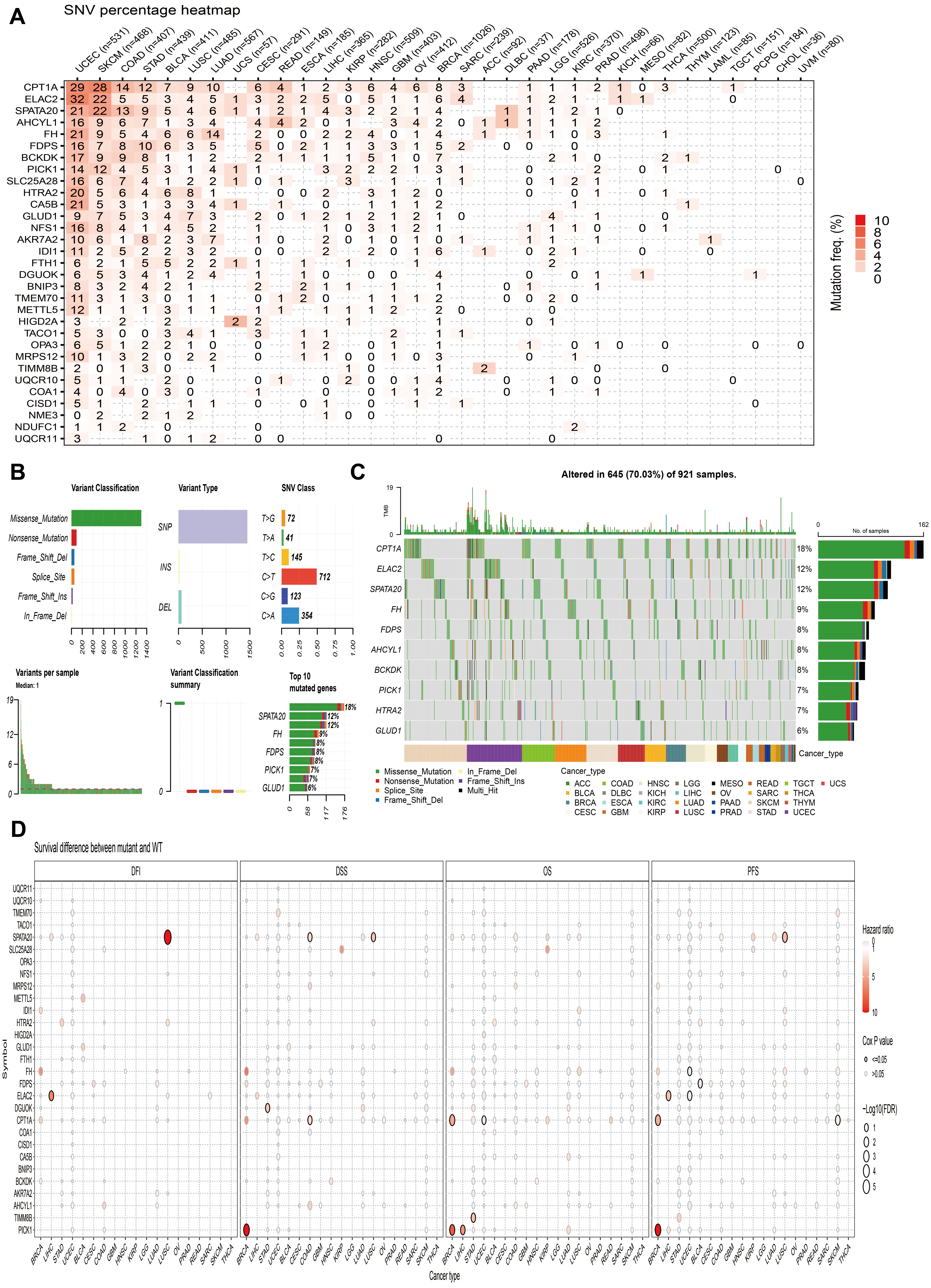

Supplement: Supplementary Figure 9 — The mutation landscape of MitoScore signature genes in pan-cancer analysis. (A) Copilot of the signature gene mutation frequency in pan cancers. (B, C) Copilot of the single-nucleotide variant in pan cancers. (D) Summarizes the survival difference between mutant and WT. [file Image9.jpeg]

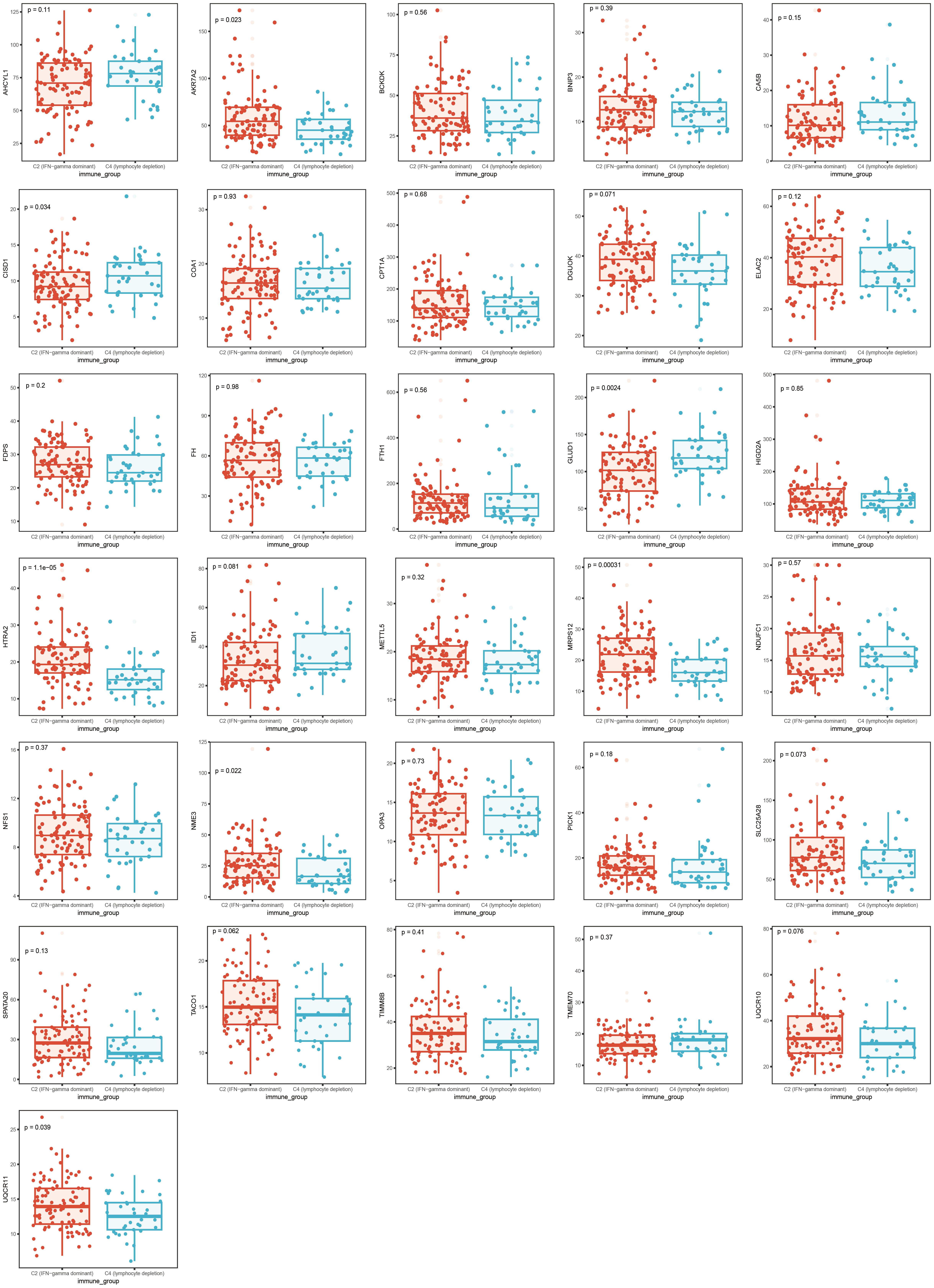

Supplement: Supplementary Figure 10 — Box plot portrays the dissimilarities in the cancer immunity subgroup between MitoScore signature genes. [file Image10.jpeg]
